# Therapeutic Reference Range for Aripiprazole in Schizophrenia Revised: a Systematic Review and Metaanalysis

**DOI:** 10.1007/s00213-022-06233-2

**Published:** 2022-10-05

**Authors:** Xenia M. Hart, Christoph Hiemke, Luzie Eichentopf, Xenija M. Lense, Hans Willi Clement, Andreas Conca, Frank Faltraco, Vincenzo Florio, Jessica Grüner, Ursula Havemann-Reinecke, Espen Molden, Michael Paulzen, Georgios Schoretsanitis, Thomas G. Riemer, Gerhard Gründer

**Affiliations:** 1 Department of Molecular Neuroimaging, Medical Faculty Mannheim, Central Institute of Mental Health, University of Heidelberg, Mannheim, Germany; 2 Department of Psychiatry and Psychotherapy and Institute of Clinical Chemistry and Laboratory Medicine, University Medical Center of Mainz, Mainz, Germany; 3 Department of Child and Adolescent Psychiatry, University of Freiburg, Freiburg, Germany; 4 Sanitario Di Bolzano, Servizio Psichiatrico del Comprensorio, Bolzano, Italy; 5 Department of Psychiatry and Psychotherapy, University of Rostock, Rostock, Germany; 6 Department of Psychiatry and Psychotherapy, University Medical Center Göttingen, Göttingen, Germany; 7 Center for Psychopharmacology, Diakonhjemmet Hospital, Oslo, Norway; 8 Department of Psychiatry, Psychotherapy and Psychosomatics, Alexianer Hospital Aachen, Aachen, Germany; 9 Department of Psychiatry, Psychotherapy and Psychosomatics, University of Zurich, Psychiatric University Hospital Zurich, Zurich, Switzerland; 10 Department of Psychiatry, The Zucker Hillside Hospital, Northwell Health, New York, NY USA; 11 Zucker School of Medicine at Northwell/Hofstra, Department of Psychiatry, Hempstead, NY USA; 12 Institute of Clinical Pharmacology and Toxicology, Berlin Institute of Health, Charité-Universitätsmedizin Berlin, Freie Universität Berlin, Humboldt-Universität Zu Berlin, Berlin, Germany; 13 Arbeitsgemeinschaft Für Neuropsychopharmakologie Und Pharmakopsychiatrie (AGNP), Work group Therapeutic Drug Monitoring, München, Germany

**Keywords:** Aripiprazole, Reference range, Blood level, Therapeutic Drug Monitoring, Clinical effects, Adverse drug reaction, Dopamine receptor occupancy

## Abstract

**Rationale:**

While one of the basic axioms of pharmacology postulates that there is a relationship between the concentration and effects of a drug, the value of measuring blood levels is questioned by many clinicians. This is due to the often-missing validation of therapeutic reference ranges.

**Objectives:**

Here, we present a prototypical meta-analysis of the relationships between blood levels of aripiprazole, its target engagement in the human brain, and clinical effects and side effects in patients with schizophrenia and related disorders.

**Methods:**

The relevant literature was systematically searched and reviewed for aripiprazole oral and injectable formulations. Population-based concentration ranges were computed (*N* = 3,373) and pharmacokinetic influences investigated.

**Results:**

Fifty-three study cohorts met the eligibility criteria. Twenty-nine studies report blood level after oral, 15 after injectable formulations, and nine were positron emission tomography studies. Conflicting evidence for a relationship between concentration, efficacy, and side effects exists (assigned level of evidence low, C; and absent, D). Population-based reference ranges are well in-line with findings from neuroimaging data and individual efficacy studies. We suggest a therapeutic reference range of *120–270* ng/ml and *180–380* ng/ml, respectively, for aripiprazole and its active moiety for the treatment of schizophrenia and related disorders.

**Conclusions:**

High interindividual variability and the influence of CYP2D6 genotypes gives a special indication for Therapeutic Drug Monitoring of oral and long-acting aripiprazole. A starting dose of 10 mg will in most patients result in effective concentrations in blood and brain. 5 mg will be sufficient for known poor metabolizers.

**Supplementary Information:**

The online version contains supplementary material available at 10.1007/s00213-022-06233-2.

## Introduction

One of the fundamental principles of pharmacology is the existence of a relationship between the dose (or concentration) of a drug and the organism’s (patient’s) response to that drug. For drugs that exert their clinical effect by binding to a receptor (or transporter), the dose–response relationship is closely related to the drug–receptor binding relationship. Since the blood levels (BLs) of orally administered drugs are extremely variable at a given dose (Gründer et al. 2008), the BL of a drug is usually a much more accurate indicator of the extent to which the molecular target is occupied by the substance. Despite the fundamental validity of these basic principles of pharmacology, *therapeutic reference ranges* for BLs of drugs are still considered by many clinicians to be insufficiently valid to guide therapy with psychotropic drugs. *Therapeutic Drug Monitoring (TDM)*, the assessment of medication BLs for personalized treatment, is primarily used as a tool to identify adherence problems or for problem solving. Here, we present a prototypic systematic review and metaanalysis on the relationship between BLs of aripiprazole (ARI), and first, clinical outcome, and second, dopamine receptor occupancy, with the aim of establishing a definitive reference range for ARI in patients with schizophrenia and related disorders.

Aripiprazole attracted particular interest when it appeared on the market because of its novel mechanism of action (Gründer et al. 2003). ARI acts as a partial agonist at D_2/3_ and 5-HT_1A_ receptors, and as an antagonist at serotonin 5-HT_2A_ receptors (Gründer et al. 2006). Its active metabolite, dehydroaripiprazole (D-ARI) has a similar pharmacological profile to its parent compound, thus is a relevant mediator for treatment outcome. ARI’s antipsychotic efficacy is comparable to that of antagonist antipsychotics. Extrapyramidal side effects and weight gain are rare, and prolactin is decreased rather than increased (Huhn et al. 2019). Clinically used ARI doses range from 10 to 30 mg daily (Otsuka Pharmaceutical Co. 2016). A recent work, however, revealed that a dose of around 12 mg/day is sufficient to produce 95% of the maximum effect of ARI in patients with schizophrenia (Leucht et al. 2020). The authors concluded that patients usually do not benefit from higher doses.

International guidelines for Therapeutic Drug Monitoring (TDM) propose a therapeutic reference range of *100–350* ng/ml for ARI and *150–500* ng/ml for the active moiety (Hiemke et al. 2018; Schoretsanitis et al. 2021). While TDM is recommended for dose titration in some patients treated with ARI, the evidence for a relationship between BLs and clinical efficacy and side effects is sparse (Sparshatt et al. 2010; Lopez and Kane 2013; Mauri et al. 2018). However, the fact that a relationship between BLs and clinical effects has not been convincingly demonstrated to date does not mean that it does not exist. The available studies may simply be methodologically inadequate (Preskorn 2013; Hiemke 2019). We consider the methodology proposed here as a prototype for establishing therapeutic reference ranges for antipsychotic drugs.

## Methods

### Inclusion Criteria

Both randomized controlled trials (RCTs) and uncontrolled studies reporting ARI blood concentrations in humans (serum or plasma), referred to herein as BLs, were eligible for inclusion, especially those investigating relationships with clinical effects or D_2/3_ receptor occupancy (suppl. table S2). Reviews and metaanalysis investigating a concentration/efficacy-relationship for ARI were also included. Studies were included regardless of ARI dosage forms. The indications were restricted to schizophrenia, schizophrenia spectrum disorders, and bipolar disorder.

### Study selection process

We followed our previously published protocol and relevant guidelines (Page et al. 2021; Hart et al. 2021) including a quality control of publications (Hart et al. 2021) and grading of available evidence (Hasan et al. 2019) (for complete search terms see suppl. S1). Risk of bias was assessed with the Cochrane risk-of-bias tool 2.0 (Sterne et al. 2019) and a previously reported rating instrument (Hart et al. 2021). Four electronic databases were systematically searched on February 16, 2021 without restriction of language or publication date (PsycINFO, Medline via PubMed, Cochrane CENTRAL, Web of Science; last updated January 31, 2022). Search terms for aripiprazole, blood concentrations, drug monitoring, PET, and SPECT were used. See supplemental material S1 for full database search strings. No preset database search filters and no restrictions regarding the publication date were applied. The search was complemented by a hand search in the reference lists of the included publications and in former published guidelines. After the removal of duplicates, screening of the literature was performed by two independent reviewers (LE, XH) according to PRISMA guidelines. In cases where a final decision on the inclusion could not be made based on the abstract alone, the full article was reviewed. Both reviewers independently extracted the following information from each study: lead author, year, title, country, study design, number and details of subjects, diagnosis, mean dose ± standard deviation (SD), mean blood concentration ± SD, concentration range, clinical efficacy or side effect measures, and main outcomes. Any disagreements between the reviewers were resolved in a subsequent discussion. Additional data were requested from the authors, whenever concentration data were not complete. This study is registered under PROSPERO number CRD42020215872.

### Qualitative and quantitative synthesis

Outcomes of interest for the qualitative synthesis were reports of an association between ARI and/or D-ARI BLs and clinical effect, either efficacy or side effects. Eligible reports could be qualitative or quantitative, continuous or categorical but required a structured clinical assessment by a rating scale. Factors influencing ARI and D-ARI BL among patients were extracted. Studies reporting D_2/3_ receptor occupancy in relation to the participants’ BLs were extracted, and 90% effective concentrations (EC_90_ values) were computed from EC_50_ as previously described (Hart et al. 2022). For the quantitative synthesis, means, standard deviations, medians, and interquartile ranges of relevant BLs were assessed. Means and standard deviations of the C/D ratio were selected. Data were either extracted from the manuscript or, if numbers for the whole sample were given, calculated manually.

### Statistical Analysis

A combined metaanalysis was performed using the R (Version 4.0.3) “metafor” and “meta” package. I^2^ statistic was used to evaluate heterogeneity of the studies, with I^2^ values > 50% indicating heterogeneity. Ninety-five percent confidence intervals (CIs) were calculated from mean concentrations and C/D value, and data were combined using random-effect models based on the I^2^ statistic. Four quality assessment criteria that could have a potential influence on the clinical validity of a therapeutic reference range were identified a priory (Q1 “ethnic group Caucasian,” Q2b “diagnosis schizophrenia,” Q4 “dose design,” and Q6a “sampling at trough”). Their impact as moderating factors on mean BLs was investigated by subgroup analyses of studies rated sufficient or insufficient on these criteria if a minimum of three records per group were available. Forest plots of subgroup differences identified as significant (p ≤ 0.05) were retrieved for visualization of subgroup differences. Linear regression analysis was used to display the relationship between ARI dose and ARI and D-ARI BLs.

## Results

### Study overview

**Fig. 1:**
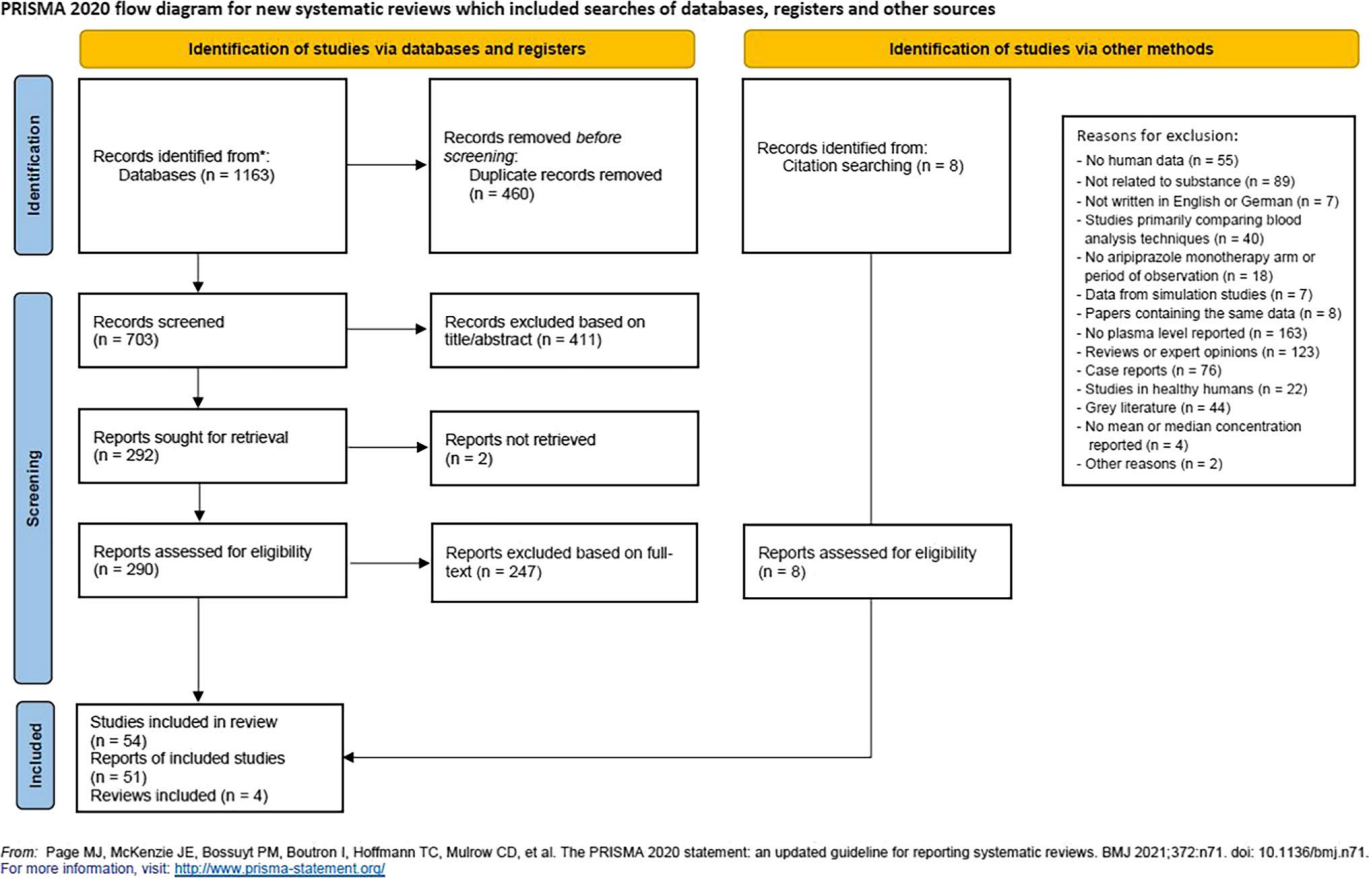
Study Overview according to PRISMA

From the 715 articles initially identified, a total of 51 articles comprising of 53 studies (Fig. 1) published from 2002 to 2021 were selected (for study details see suppl. table S3–S5). Four articles reported results from two or more separate patient samples including one article that developed a population-based pharmacokinetic model. In total, 29 studies were identified that report BL after oral ARI administration. Thirteen of them additionally reported results from clinical efficacy or side effect assessments. Of 15 studies that reported BL after ARI injections (13 LAI, 2 acute), nine studies reported clinical efficacy measures. Nine neuroimaging studies on (striatal) D_2/3_ receptor occupancy were found. Rating results are presented in the supplemental material S6-S11.

### Risk of bias rating for TDM component

See suppl. fig. S6 and S11 for results. The most frequently missed TDM criterion was Q1 “study population,” since the majority of studies did not solely include Caucasian patients. The second most frequently missed criteria were comedication (Q3) and dose design (Q4) followed by an inhomogeneous diagnosis (Q2b). More than half of the studies used a naturalistic design allowing for flexible dosing or administered single doses. As a result of a high percentage of uncontrolled cohort and retrospective TDM studies, comedication with psychotropic and pharmacokinetically interfering drugs was common among studies. Studies with retrospective data collection, such as cross-sectional studies, could usually not fulfill the criterion of a predefined sampling schedule (Q7a). However, even among cohort studies, single sampling was common. Nevertheless, most studies reported sufficiently broad concentration ranges for ARI (Q7b), a crucial qualification to find a concentration/efficacy-relationship. Most studies selected patients according to the psychiatric classification system “Diagnostic and Statistical Manual of Mental Disorders version IV or 5” (Q2a). However, studies often did not distinguish between patients with a diagnosis of schizophrenia and with other psychotic disorders (Q2b). The analytical method (Q5) was rated as insufficient in 16 studies because precise information on the detection limit was missing. Sampling time (Q6b) and steady state (Q6a) were given in the majority of selected studies.

### Concentration/efficacy-relationship

In general, we found highly heterogeneous reports of clinical efficacy/concentration-relationships (Table 1). A clear relationship between ARI BL and antipsychotic effects was reported by two prospective cohort studies, both considered of having moderate risk of bias (TDM score; 4/10 and 8/10, ST score; both 6/10) (Lin et al. 2011; Nemoto et al. 2012). One study, however, introduced a considerable amount of bias by add-on therapy with the antidepressant and CYP2D6 inhibitor paroxetine (Nemoto et al. 2012). Another study by Lin et al (2011) in patients with schizophrenia or schizoaffective disorder with an acute exacerbation, the only study that a priori aimed at finding a concentration/efficacy-relationship, did not allow for relevant psychiatric comedication (Lin et al. 2011). After six weeks of treatment under flexible dosing, responders, defined by at least 20% decrease in PANSS total score, had higher D-ARI and AM BLs than nonresponders (however not significant for ARI alone). Nakamura and colleagues (2009) reported conflicting results in patients with SCZ, which should, however, also be regarded with caution due to the combination with low doses of the anticonvulsant drug carbamazepine (which lowers ARI levels by inducing CYP3A4) (Nakamura et al. 2009). In addition, one study in patients with schizophrenia, other psychotic disorders or bipolar disorder, reported better attention and working memory in patients with higher ARI BLs (Steen et al. 2017). Another study reported a negative association between patient-reported physical well-being and very high D_2/3_ receptor occupancy, estimated from ARI BLs in patients with schizophrenia (Veselinović et al. 2019). No metaanalysis on the concentration/effect-relationship of ARI is available. None of the LAI studies has aimed at or described a correlation between ARI BLs, response, or side effects. Concomitant oral antipsychotic treatment was given in all LAI studies that included patients with schizophrenia. To sum up, despite conflicting results from pharmacokinetic studies, one study at moderate risk of bias was able to report a positive association between ARI concentration and clinical efficacy, which justifies the classification of the evidence as “low” for the concentration/efficacy-relationship after oral administration (Level C; low) (Hart et al. 2021).

**Table 1:** Level of evidence; Summarized results of the qualitative synthesis. Studies reporting a concentration/efficacy- or side effect-relationship

Reference	Design and subjects	Efficacy	Side effects	BL range	Psyc. Comed	TDM Score	Study score	Comment and risk for Bias
Nemoto et al. 2012	Prospective CS; paroxetine add-on; fixed ARI doses (mean 14.6 mg); SCZ; *N* = 14	Positive (CGI)	Not found (DIEPS)	Yes	Yes	8/10	6/10	CGI decreased with increasing ARI BL; CAVE: add-on
Lin et al. 2011	Prospective CS with flexible doses (mean 15 mg/day); SCZ or SAD; *N* = 45	Positive (PANSS)	Not found (AIMS, BARS, SAS)	Yes	No	6/10	4/10	Higher ARI BL in responders (20% decrease in PANSS score)
Nakamura et al. 2009	Prospective CS with fixed doses (mean 22 mg/day); carbamazepine add-on; SCZ; *N* = 18	Negative (PANSS)	Positive (UKU)	Yes	Yes	8/10	4/10	Higher response and less neurological AEs with decreasing ARI BL; CAVE: add-on
Hwang et al. 2015	Cluster RCT with fixed doses (15 mg/day); SCZ or SAD; *N* = 79	Not available	Negative (BARS; Akathisia)	No	Yes	7/10	Moderate	Higher ARI BL correlated with greater reduction in BARS on day 56
Steen et al. 2017	CSS; flexible doses, multiple diagnoses; *N* = 373	Not available	Not available	Yes	No	5/10	6/9	Better attention and working memory nominally associated with higher ARI BL
Veselinovic et al. 2019	Cohort nested in RCT; flexible design (mean 15.5 mg/day); SCZ; *N* = 11	Not available	Not found (SAS, BARS, AIMS)	Yes	Yes	8/10	6/10	Physical and mental well-being correlated negatively with estimated D_2_ receptor occupancy

BL = blood level, CS = cohort study, CSS = cross-sectional study, Psyc. Comed. = psychiatric comedication, RCT = randomized controlled trial, SCZ = schizophrenia, SAD = schizoaffective disorder

### Concentration/side effect-relationship

A total of ten studies measured general or specific motor side effects using a structured clinical rating scale. Five studies did not detect an association between BLs and side effects. One study found a general decrease in neurological side effects (assessed by the UKU side effect rating scale) when ARI BLs decreased after carbamazepine add-on therapy in patients with schizophrenia (Nakamura et al. 2009). As discussed above, this finding should be treated with care, because carbamazepine exerts psychotropic effects itself. In a cluster RCT, Hwang et al. (2015) observed that after 56 days of treatment, the sample of schizophrenia and schizoaffective disorder patients with higher ARI BLs scored lower on an akathisia scale (Hwang et al. 2015). This counterintuitive result, however, could also be interpreted as a manifestation of the positive effect of ARI on psychomotor agitation with continued therapy. Of note, all patients had BLs within the currently recommended reference range of ARI (*100–350* ng/ml). The study was rated with a moderate risk of bias (TDM score; 4/10, RoB some concerns). No systematic review or metaanalysis on the concentration/side effect-relationship is available. Overall, the available evidence on side effects caused by ARI treatment, i.e., mainly psychomotor related events such as akathisia, does not support a causal relationship with BLs. A possible relationship could, however, been obscured by rather unspecific instruments that were used to assess potential medication-related side effects. The existing studies do not allow for an evaluation (Level D; no evidence).

### Dopamine receptor occupancy

Five positron emission tomography studies were identified that provide valuable insights into the association between ARI blood concentrations and striatal D_2/3_ receptor occupancy (Table 2 (Hart et al. 2022)). Three out of four studies that included patients with schizophrenia additionally measured clinical effects (Mamo et al. 2007; Kegeles et al. 2008; Shin et al. 2018). Overall, a high target engagement of D_2/3_ receptors (> 90%), a prerequisite for partial agonist antipsychotic efficacy (Hart et al. 2022), was reached with ARI BLs of 90 ng/ml (putamen; patients with schizophrenia) (Gründer et al. 2008), 100 ng/ml (striatum; healthy volunteers) (Kim et al. 2012), and 110 ng/ml (putamen; healthy volunteers) (Takahata et al. 2012), and 180 ng/ml for the AM (putamen; patients with schizophrenia) (Gründer et al. 2008). After fixed doses of ARI, one study reported an ED_80_ value of 6 mg (Kegeles et al. 2008). The authors found a decrease in PANSS positive subscale scores with higher target engagement (*N* = 7). Another study could not confirm this finding but reported extrapyramidal side effects (EPS) in two patients with very high BLs and a D_2_ receptor occupancy > 90% (Mamo et al. 2007; Mizrahi et al. 2009). To sum up, PET studies suggest a strong relationship between target engagement and BL with ARI concentrations above 90 ng/ml resulting in clinically effective target engagement.

**Table 2:** Selected dopamine receptor occupancy studies that report a relationship between ARI BL or dose and D_2_ occupancy. EC_90_ estimated from EC_50_

*Reference*	Design and subjects	PET tracer	Mean ARI dose (range) [mg/day]	Mean ARI BL (range) [ng/ml]	Mean receptor occupancy (%)	EC_50_ [ng/ml]	EC_90_ [ng/ml]	Comment
Kim et al. 2012	RCT; *N* = 18; healthy volunteers; mean age 23; 100% males	[^11^C]raclopride	13 ± 12 (2–30)	Peak: 3.4 ± 0.9 per mg	D_2/3_: 62 ± 21 (s)	11.1 (s)	100 (s)	Values reported for PK model; PK/PD model estimates EC_90_ of 77 ng/ml (s).
Gründer et al. 2008	CS; *N* = 16/8 (medicated/ medication-free); SCZ or SAD (DSM-4); mean age 30; 94% males	[^18^F]fallypride	19 ± 7 (5–30)	245 ± 307	D_2/3_: 83 ± 1 (p), 84 ± 1 (c)	10 ± 4 (p) 9 ± 4 (c)	90 (p), 81 (c)	Complete occupancy with ARI BL > 100–150 ng/ml. EC_90_ for AM is 180 ng/ml.
Takahata et al. 2012	CS; *N* = 11; healthy volunteers; mean age 24 ± 4; 100% males	[^11^C]raclopride, [11C]FLB457	6	29 ± 5	D_2/3_: 74 ± 7 (c), 70.1 ± 6.3 (p)	9.9 (s), 12.2 (p)	89 (s), 110 (p)	Concentration reported for raclopride scans; lower in FLB457. No preferential extrastriatal binding of ARI.
Mamo et al. 2007 ; Mizrahi et al. 2009	RCT; *N* = 12; SCZ or SAD; mean age 31; 75% males	[^11^C]raclopride, [18F]setoperone, [^11^C]WAY100635	19 ± 8 (10–30)	221 ± 179	D_2/3_: 87 ± 4 (p), 92.9 ± 5.7 (c)	NA	NA	ARI and DARI BL correlated with D_2_ occup. (p and s). No corr. between occup. And clinical or well-being scores. EPS in 2 patients with occupancy > 90%.
Kegeles et al. 2008	CS; *N* = 19; SCZ or SAD (DSM-4); mean age 29; 79% males	[^18^F]fallypride	14 ± 11 (2–40)	NA	D_2/3_: NA 80 ± 15 (s) in 15 mg	ED_80_ 5.6 ± 1.0 (s) ~ 100	NA	Dose correlated with ARI BL, PANSS positive scale correlated with D_2_ occup. (s). No EPS occured.

c = caudate, CS = cohort study, EPS = extrapyramidal side effects, NA =. Not available, p = putamen, RCT = randomized controlled trial, SCZ = schizophrenia, SAD = schizoaffective disorder, s = striatum

### Population-based target concentration range

#### Blood level after fixed and flexible dosing

**Fig. 2:**
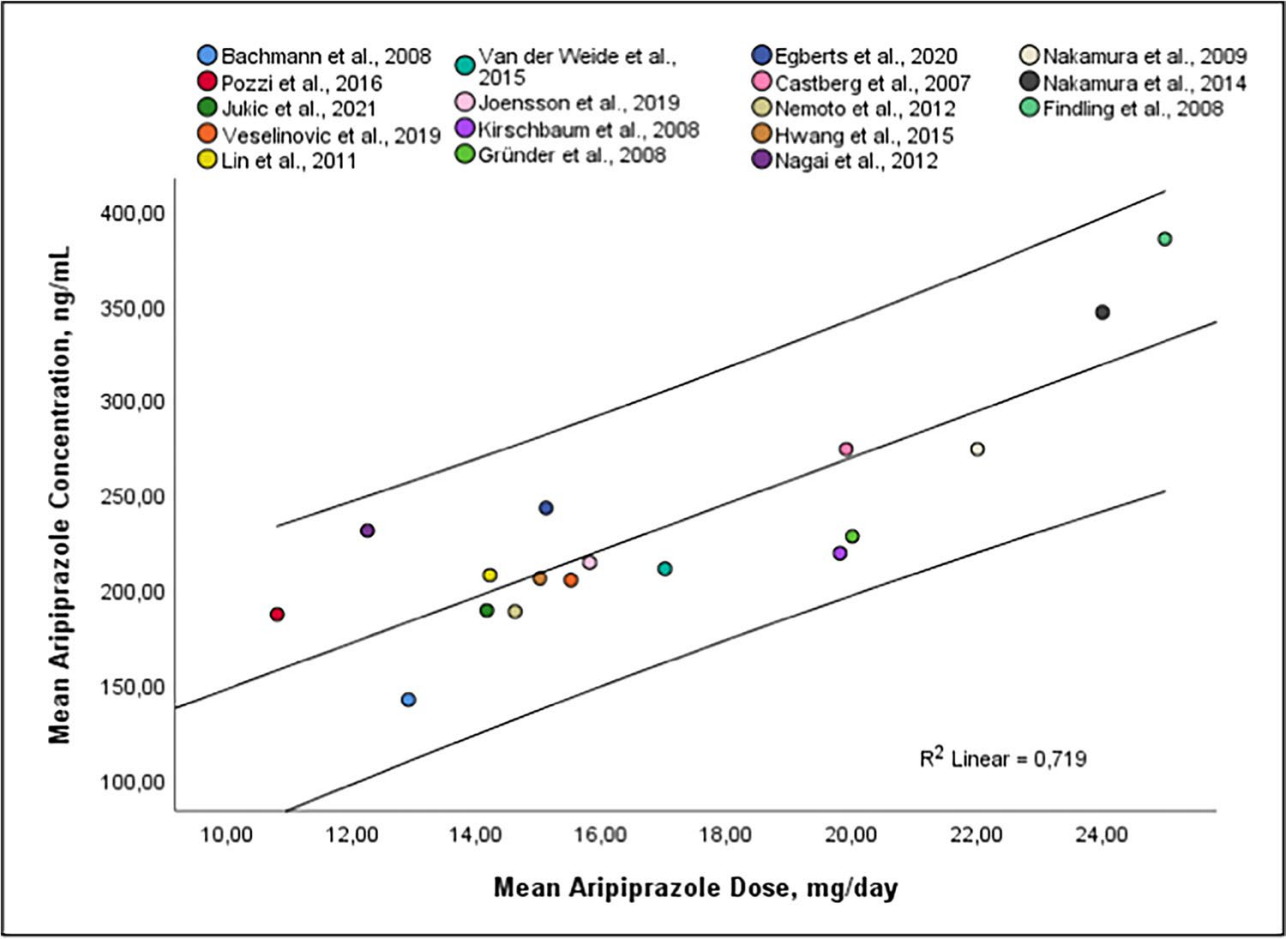
Mean Aripiprazole Dose [mg/day] Versus Mean Aripiprazole blood concentration [ng/ml] (β-coefficient = 12.205 (8.007–16.403), r2 = 0.719, P < .0001, y = 25.612 + 12.205 * x) N= 3,778

**Fig. 3:**
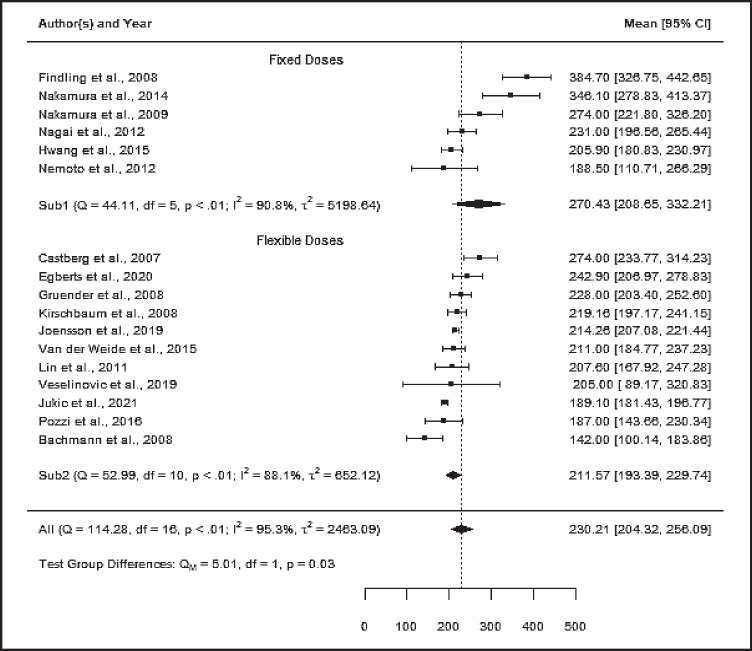
Overall mean ARI concentration estimate [ng/ml] with subgroup analysis „dose design,”( *N* = 3,778)

Studies were excluded in case of insufficient data reports, and one study each due to i) sole inclusion of patients with bipolar disorder, ii) sampling at peak, and iii) unusual dose regimen. Linear regression analysis of mean concentrations across 17 and 10 studies show a strong relationship between dose and ARI concentration (N= 3,778, *r* = *0.85, P* < *0.0001*, Fig. 2) and between dose and the AM concentration (N= 3,280, *r* = *0.79, p* = *0.007*, suppl. fig. S12). The combined mean C/D ratio across seven and six studies was 13.8 (ng/ml)/(mg/day) [12.4, 15.3] (*Q* = *38.1, df* = *6, p ≤ 0.05, I*^*2*^ = *88%, T*^*2*^ = *2.96*) and 18.2 [16.6, 19.7] *(Q* = *29.3, df* = *5, p* < *0.0001, I*^*2*^ = *84%, T*^*2*^ = *2.81*) for ARI and the AM, respectively ( Table 3). The combined mean concentration across 17 and nine studies was 230 ng/ml [204, 256] *(N* = *3778)* and 305 [257, 353] (*N* = *3332, Q* = *84.8, df* = *9, p* < *0.01, I*^*2*^ = *98%, T*^*2*^ = *5205*) for ARI and the AM, respectively (suppl. fig. S13). Mean doses were 17 and 16 mg/day. Subgroup analysis could be performed accordingly with all four predefined quality assessment criteria, since at least three studies per subgroup were available (suppl. table S14). One subgroup comparison “dose design” revealed significantly differing mean drug concentrations between both groups (*Chi*^*2*^ = *5.0, df* = *1, p* = *0.03, I*^*2*^ = *94%*). Studies using fixed dose designs used higher doses resulting in higher drug concentrations compared to studies comprising real-world patients from psychiatric clinics (Fig. 3).

**Table 3:** Expected concentration ranges from approved doses based on our findings (C/D ratios) and based on ratios from TDM Guidelines

Administered Dose [mg/day]	Expected ARI BL [ng/ml] based on C/D ratio 13.82	Dose-related range based on TDM Guidelines 11.72	Expected ARI + D-ARI BL [ng/ml] based on C/D ratio 18.18	Dose-related range based on TDM Guidelines 16.45
5	69.1 [62.0, 76.3]	58.6 [41.8–76.5]	90.9 [83.21, 98.7]	82.3 [56.0–109.5]
10	138.2 [123.9, 152.5]	117.2 [81.5–152.9]	181.8 [166.3, 197.3]	164.5 [111.9–218.9]
20	276.4 [247.8, 305]	234.4 [163.0–305.8]	363.6 [332.6, 394.6]	329 [223.8–437.8]
30	414.6 [371.7, 457.5]	351.6 [244.5–458.7]	545.4 [498.9, 591.9]	493.5 [335.7–656.7]

#### Concentration range from real-world patients

**Fig. 4:**
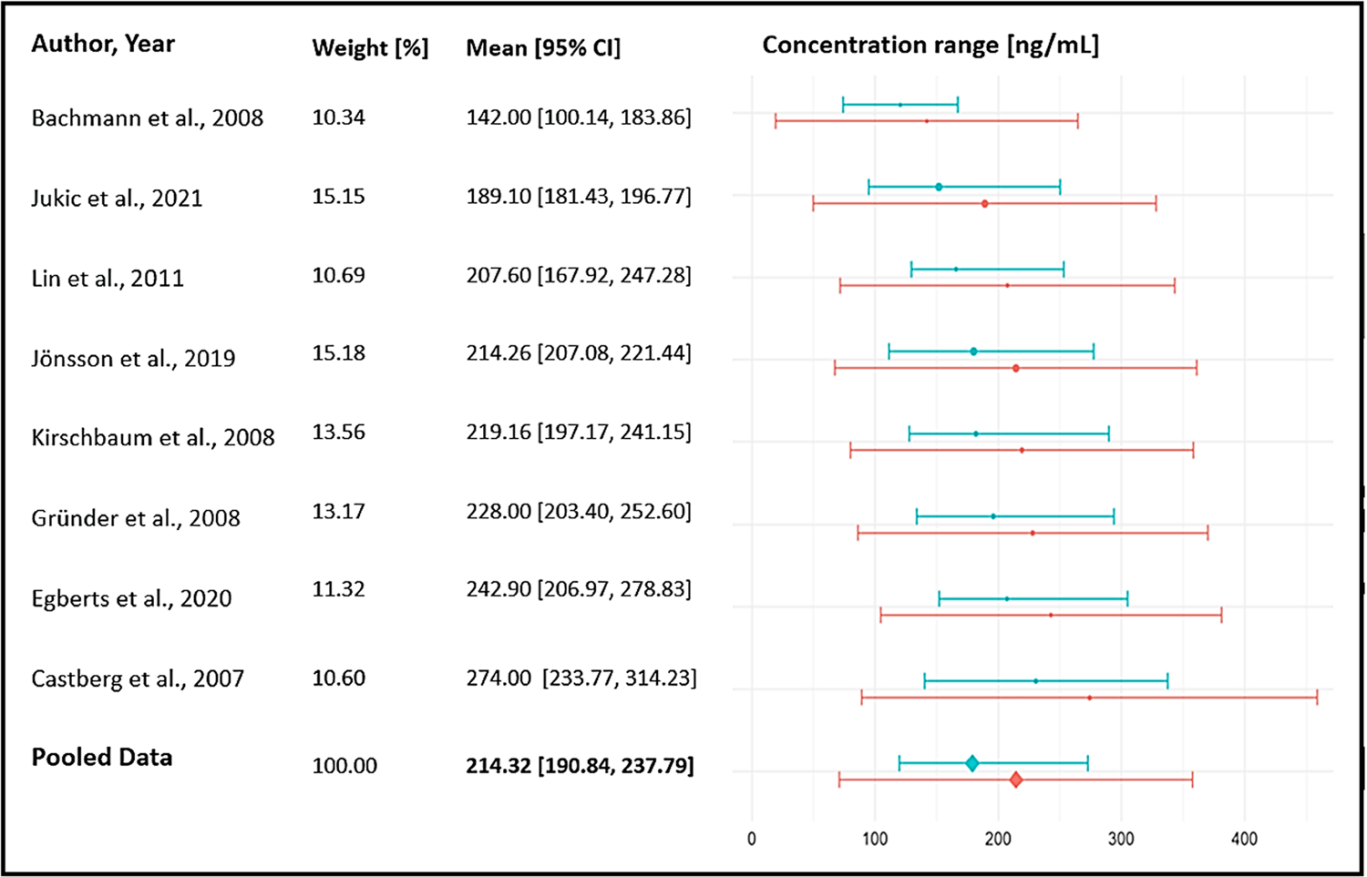
Target ranges for ARI [ng/ml] (N= 3,778, Combined range mean ± SD: 71–358, combined interquartile range: 120–273, mean concentration 214 [191, 238] (Q = 52.12, df = 7, p < .0001, I^2^ = 93.2, Ƭ^2^ = 932.1))(Mean ± SD ranges of studies depicted as red lines, 25^th^–75^th^ interquartile ranges of studies depicted as blue lines.)

Data from 3,373 patients with schizophrenia and schizophrenia spectrum disorders that were treated with oral ARI under flexible dosing were derived from eight studies using a naturalistic design. Two preliminary ranges were computed i) a mean ± standard deviation (SD) range of *71–358* ng/ml and ii) a 25^th^–75^th^ interquartile range of *120–273* ng/ml (Fig. 4). Two studies in children and/or adolescents discussed the comparability of the results with those obtained from adults (Bachmann et al. 2008; Egberts et al. 2020).

#### Factors influencing ARI blood levels

##### Sex, age, and body weight

Three studies reported significantly higher BLs in females compared to males (Table 4). Linear regression analysis with correction for dose, weight, age, and comedication revealed that girls had about 41% higher BLs than boys (Egberts et al. 2020). Another study found dose-corrected BLs about 10% higher in women (Jönsson et al. 2019). One conflicting result was reported by a study that found 28% higher mean ARI concentrations corrected for defined daily doses (DDD) in men than in woman (Hoekstra et al. 2021). Five studies, including two studies that used advanced modeling techniques, did not find sex-related differences in BLs. Of eight studies that investigated age or age groups in relationship to BLs, only two studies found a weak correlation. In a large naturalistic dataset (*N* = *1,610, age 8–92 years*), 16% higher dose-corrected concentrations were noted in patients older than 65 years. Most of the remaining studies did not include patients older than 65 years. Four studies consistently found no association between body weight and ARI BLs.

**Table 4:** Factors influencing ARI blood levels after oral administration (Y = correlation found * < .05, ** < .001, p < 0.0001***; (Y) = trend found, not significant or only in discussion; N= no correlation or trend found; blank = not reported)

No	Reference	Dose (linear)	CYP2D6 Genotype	Sex (higher in female)	Age	Body weight	Comedication (CYP2D6 or − 3A4)	
1	Pozzi et al. 2016	Y**			N	N	Y**	r = 0.37 (Number)
2	Egberts et al. 2020	Y**		Y**	(Y*)	N	(Y)	
3	Kirschbaum et al. 2008	Y**					Y*	CYP2D6
4	Lin et al. 2011	Y***						
5	Molden et al. 2006	Y***		N			N	
6	Jönsson et al. 2019	(Y)		Y***	Y*			
7	Veselinovic et al. 2019	(Y*)		(Y)				
8	Steen et al. 2017	Y**						
9	Gründer et al. 2008	Y**						
10	Van der Weide et al. 2015	Y*	Y*	N	N			
11	Kim et al. 2008		Y**	N	N	N		
12	Hwang et al. 2015		Y*					
13	Nemoto et al. 2012		Y*				Y*	Paroxetine
14	Nagai et al. 2012		Y**	Y**	N			
15	Nakamura et al. 2014		Y*				N	Haloperidol
16	Hendset et al. 2007		Y*					
17	Jukic et al. 2019		Y*					
18	Nakamura et al. 2009		N				Y**	Carbamazepine
19	Nemoto et al. 2014		N				Y*	Paroxetine
20	Hoekstra et al. 2021			Y*				
21	Bachmann et al. 2008			N	N	N	N	
22	Zuo et al. 2006						N	Clozapine
23	Castberg et al. 2007			N	N		(Y)	
24	Eryilmaz et al. 2014						Y*	Valproate
25	Waade et al. 2009						Y*	CYP2D6, CYP3A4

##### Concomitant Medication

Most studies that were interested in the effect of comedication measured drug concentrations before and after the add-on of a pharmacokinetically relevant drug. Two studies showed an increase of ARI BLs after the administration of paroxetine (Nemoto et al. 2012, 2014) (Table 4). The mood stabilizers carbamazepine and valproate were found to decrease ARI (AM) BLs by 65% and 23%, respectively (Nakamura et al. 2009; Eryilmaz et al. 2014). No influence of escitalopram (Nemoto et al. 2014), haloperidol (Nakamura et al. 2014) or clozapine (Zuo et al. 2006) coadministration was found. Concurrent treatment with *CYP3A4* inducers, *CYP2D6* inhibitors, alimemazine, or lithium changed BLs by 40%–60%, which has to be considered clinically relevant (Waade et al. 2009). Similar effects were shown in children and adolescents (Kirschbaum et al. 2008; Pozzi et al. 2016).

##### CYP2D6 Genotyping

Ten studies investigated whether the relationships of the genetic variants of *CYP2D6* with ARI BLs are consistent with known functions (phenotypes) (Table 4; supplemental S15 for phenotype classifications). Eight studies reported an association of *CYP2D6* phenotypes with BLs whereas two studies could not confirm these findings. One study in Asian patients reported lower ARI BL with CYP2D6*10 (vt) alleles (intermediate metabolizers, IM) (Hwang et al. 2015). This finding was confirmed in another study (Nemoto et al. 2012). The same group was not able to replicate this result (Nemoto et al. 2014). A Japanese study found that dose-corrected ARI and AM concentrations increased with a general increase in the number of the mutated CYP2D6 alleles *5, *10, and *14 (Nagai et al. 2012). A Norwegian study reported 50% higher median BLs in *CYP2D6* poor metabolizers (PM) than in extensive metabolizers (EM) (Hendset et al. 2007). Two studies performed more comprehensive classifications of phenotypes with subjects classified into four groups. A Swedish study found an increase in AM concentrations by about 40% in PMs and IMs (Jukic et al. 2019). A Dutch study performed a multiple regression analysis and found dose and predicted CYP2D6 phenotype as influencing factors on ARI and D-ARI BLs (*r*^*2*^ = *0.01*) (van der Weide and van der Weide 2015). Dose-corrected concentrations were 56% higher in predicted PMs, 4% higher in IMs and 11% lower in ultrarapid metabolizers (UMs) compared to EMs. A similar result was replicated in a study that has used pharmacokinetic modeling methods to explain interindividual variance in BLs (Kim et al. 2008). *CYP2D6* genotype, but not sex, age or bodyweight, remained a significant covariate in the final model. 1.5–1.7-fold higher BLs in PMs and IMs were also found in patients after LAI treatment (Tveito et al. 2020).

#### TDM for long-acting injectable (LAI) aripiprazole

##### Aripiprazole lauroxil (AL)

Three randomized studies assessed pharmacokinetic profiles after single injections of AL and two studies applied multiple injections. As described previously, higher peak plasma concentrations were found following administration to the deltoid site when compared with the gluteal site (Hard et al. 2019; Schoretsanitis et al. 2021). All patients were stabilized on oral antipsychotic treatment; clinical ratings remained stable. After five gluteal injections of 441 (q4wk), 882 (q6wk), or 1064 (q8wk) mg, patients showed quite similar average ARI concentrations (*126–141* ng/ml). Maximum concentrations were below 200 ng/ml for all dosages (Hard et al. 2017). Before reaching steady state, after 12 weeks, the median BLs only exceeded the 120 ng/ml threshold at the high dosages of 662 and 882 mg (q4wk), not at the 441 mg dosage nor at longer application periods (Hard et al. 2018). However, over the time course of a year, simulated median BLs in all dosage regimens would hit the threshold.

##### Aripiprazole monohydrate (AM)

Three studies (two RCTs, one observational study) report ARI BLs after multiple injections of AM 200, 300, or 400 mg (q4wk) for up to one year. In patients with schizophrenia, clinical scale scores remained stable under oral antipsychotic treatment. After five injections of 400 mg, 300 mg, and 200 mg, trough BLs were 212 ± 113/ 239 ± 133 ng/ml, 156 ± 68 ng/ml, and 95 ± 86 ng/ml (Mallikaarjun et al. 2013; Raoufinia et al. 2017). Lower BLs were found in patients with bipolar disorder after doses of 300 and 400 mg (113–132 ng/ml) (Mauri et al. 2020). The authors discussed a limit below *150 ng/ml* as therapeutic threshold for depressive and positive symptoms. In conclusion, monthly injections (e.g., five or more) of 300 mg and more will most likely result in BL above *120 ng/ml*.

## Discussion

Aripiprazole has been proven effective for the treatment of schizophrenia (Leucht et al. 2012). However, our qualitative synthesis revealed a low quality of evidence for an association between drug blood concentration and efficacy. We identified various reasons why trials were not able to find a relationship between drug concentrations and antipsychotic treatment efficacy (i.e., psychiatric comedication and flexible dose design). Only one study was able to find a clear relationship between increasing AM concentrations and antipsychotic response (PANSS scores) in patients with schizophrenia or schizoaffective disorders (Lin et al. 2011). Controlled randomized studies that aimed at finding a concentration/efficacy-relationship for ARI are almost missing. The few controlled studies that are available are of moderate to high risk for bias.

In agreement with previous reports (Citrome 2006), the present work also shows that there is no evidence for concentration-dependent side effects. There is some evidence to suggest a link between BLs and neurological side effects, particularly akathisia. However, the available clinical instruments (e.g., Barnes Akathisia Rating Scale, BARS) do not appear to be sensitive enough to distinguish between positive treatment effects (reduction in psychomotor agitation) and reduction in true akathisia (Hwang et al. 2015). The low incidence of EPS and other side effects despite high striatal D_2_ receptor occupancy in PET studies is fully consistent with the mechanism of action of ARI (Grunder et al. 2003). Even with 100% receptor occupancy, the postsynaptic signal will be sufficient to limit neurological side effects in most patients (Mizrahi et al. 2009). When reports on clinical efficacy are rare, a point of futility, meaning a concentration threshold above which a further increase in clinical efficacy cannot be expected, has been suggested as upper orienting limit for a therapeutic reference range (Meyer and Stahl 2021). To date, a clear cutoff for the onset of therapeutic response or side effects has not been shown for ARI. The present work demonstrates how population-based ranges can be used to supplement clinical efficacy data in a meaningful manner and how to identify a therapeutic reference range for a psychotropic drug from manifold types of studies, despite a low grade of first level evidence.

### Therapeutic reference range for aripiprazole

Fifty percent of patients with schizophrenia and related disorders treated under effective doses present ARI concentrations between *120* and *273* ng/ml, which is quite consistent with previously reported ranges from responders in single studies (*134–271* ng/ml based upon PANSS scores (Lin et al. 2011) and *124–286* ng/ml based upon CGI assessments (Kirschbaum et al. 2008)). In support, PET studies demonstrate consistently that therapeutically effective target engagement can be already reached with BLs around *90–110* ng/ml (*180* ng/ml for the AM) (Hart et al. 2022). The “average” patient will attain the efficacy threshold of 120 ng/ml with a dose of 9 mg once daily. The upper limit of *270* ng/ml will be reached with a dose of 20 mg/day (Table 3). For LAI formulations, AM and AL, doses of at least 300 mg and 463 mg are expected to lead to BLs within the proposed range.

### Moderating factors and implications for TDM

As a prerequisite for dose titration, the present work confirms a linear dose/concentrationrelationship for ARI within the common dosing range of 5–30 mg daily. The steady-state concentration of the major active metabolite, D-ARI, represents about 40% of the parent drug (metabolite-to-parent compound ratio (MPR); *0.40* = *(304.6–218.1)/ 218.1 ng/ml*; suppl. fig. S13). Current guidelines report dose-corrected concentration values of *11.7* and *16.5 (ng/mg)/(mg/day)* for ARI and the AM, respectively. We found somewhat higher mean C/D ratios of *13.8* and *18.2*, respectively. The findings of higher dose-corrected concentrations in our study might be explained by a higher percentage of female patients, a higher mean age, and the permission for using potentially CYP-inhibiting comedication in the included studies compared to, e.g., phase-I studies. Future research is needed to evaluate sex- and age-specific dosing. Body weight is frequently discussed in studies to explain BL differences between Asian and European study populations. However, while CYP expression patterns are certainly different among Asian and European populations, no study has systematically explored ethnic differences in ARI’s metabolism. Also, it is not clear yet, whether a different proportion of the AM relative to the parent compound leads to a change in pharmacodynamics of the drug. More eminent, higher mean BLs have consistently found in *CYP2D6* poor metabolizers. The evidence across the genetic variants of CYP2D6 is striking and calls for a dose adaption of at least 50%, which is currently not taken into account in relevant guidelines (recommended starting dose 10 mg/day for PMs) (Swen et al. 2011). Regarding clinical TDM practice, the evidence suggests that small differences in sampling time points of a few hours (i.e., 9–14 h vs. 20–24 h) may only marginally change the expected ARI blood concentration (Korell et al. 2018). An efficacy of lower doses in maintenance treatment compared to acute therapy has been discussed by dose/efficacy-metaanalysis for antipsychotic drugs (Uchida et al. 2011; Leucht et al. 2021). In the present work, studies have been included irrespective of former treatment duration. It remains unclear, if this may affect the clinical transferability of the suggested reference range.

## Conclusion

We suggest a therapeutic reference range of *120–270 ng/ml* and *180–380 ng/ml*, respectively, for ARI and its AM for the treatment of schizophrenia and related disorders. Based on the available data, the evidence for a concentration/effect-relationship is low, which results in limited implications for dose titration within the presented reference range. However, concentrations above the lower limit of the therapeutic reference range seem likely to increase treatment response. Concentrations above the upper limit are unlikely to further improve treatment response, but the incidence of adverse events seems equally unlikely to increase. A starting dose of 10 mg/day will result in effective concentrations in blood and brain of most patients. High interindividual variability and the influence of *CYP2D6* genotypes represents a special indication for TDM of oral and long-acting ARI. A starting dose of 5 mg/day might be sufficient in known *CYP2D6* PM.

## Supplementary Information

Below is the link to the electronic supplementary material.

Supplementary file1 (DOCX 845 KB)

## Data Availability

Generated Statement: The original contributions presented in the study are included in the article/supplementary material; further inquiries can be directed to the corresponding author/s.

## Abbreviations

AIMS: Abnormal Involuntary Movement Scale
AM: Active moiety, sum of ARI and DARI
ARI: Aripiprazole
BARS: Barnes Akathisia Rating Scale
BD: Bipolar disorders
BL: Blood level
C/D: Concentration to dose (mean C / mean D)
CGI: Clinical Global Impression
CGI-I: Clinical Global Impression—Improvement
CGI-S: Clinical Global Impression—Severity
CS: Cohort study
CSS: Cross-sectional study
CYP: Cytochrome P450
d: Day
D-ARI: Dehydroaripiprazole
DIEPS: Drug-induced extrapyramidal symptoms
DSM-5: Diagnostic and Statistical Manual of Mental Disorders, 5th edition
EPS: Extrapyramidal side effects
HPLC with UV detection: High-performance liquid chromatography method with UV-absorbance detection
HV: Healthy volunteers
ICD-10: International Statistical Classification of Diseases and Related Health Problems 10th edition
LC/MS/MS: Liquid chromatography/ tandem mass spectrometry
LOD: Limit of detection
LOQ: Limit of quantification
m: Month
MPR: Metabolite to parent ratio
NA: Not available
PANSS: Positive and Negative Syndrome Scale
PD Comedication: Concomitant psychotropic medication with antipsychotic efficacy
PM: Poor metabolizers
QA: Result of the study-type specific quality assessment
RCT: Randomized controlled trial
SAD: Schizoaffective disorder
SAS: Simpson-Angus Extrapyramidal Symptoms Scale
SC: Serum concentration
SCZ: Schizophrenia
SD: Standard deviation
ST score: Study-specific quality assessment score
TDM: Therapeutic Drug Monitoring
TDM score: Quality assesssment score of the Therapeutic Drug Monitoring component
UKU: UKU side effect rating scale
UPLC-MS/MS: Ultra-performance liquid chromatography–tandem mass spectrometry
w: Week
