# Supplementary material for: Therapeutic Reference Range for Aripiprazole in Schizophrenia Revised: a Systematic Review and Metaanalysis
Source: Psychopharmacology (Berl). 2022 Oct 5;239(11):3377–91. doi: 10.1007/s00213-022-06233-2 (PMC9584998; doi:10.1007/s00213-022-06233-2)
Supplement: Supplementary file 1 — Supplementary file1 (DOCX 845 KB) [file 213_2022_6233_MOESM1_ESM.docx]

***Supplement “Therapeutic Reference Range for Aripiprazole Revised:***

***a Systematic Review and Metaanalysis”***

# ***S1. Full database search strings.***

| PubMed |
| --- |
| ("aripiprazole"[MeSH Terms] OR "aripiprazol*"[Text Word] OR "abilify"[Text Word] OR "opc14597"[Text Word])  AND ("serum level*"[tw] OR "plasma level*"[tw] OR "blood level*"[tw] OR "drug level*"[tw] OR "serum concentration*"[tw] OR "plasma concentration*"[tw] OR "blood concentration*"[tw] OR "drug concentration*"[tw] OR "Drug Monitoring"[Mesh] OR "drug monitor*"[tw] OR “positron emission tomography”[MeSH Terms] OR "Positron Emission Tomogra*"[tw] OR "PET scan*"[tw] OR "Tomography, Emission Computed, Single Photon"[Mesh] OR "Single Photon Emission*"[tw] OR "SPECT"[tw] OR "CAT Scan"[tw] OR "single photon emission computed tomography computed tomography"[MeSH Terms]) NOT ("Animals"[MeSH Terms] NOT "humans"[MeSH Terms]) |
| Web of Science Core Collection |
| (TS= aripiprazole OR TS=” aripiprazole*” OR TS=abilify) AND  (TS =(serum NEAR/1 level*) OR TS = (plasma NEAR/1 level*) OR TS= (blood NEAR/1 level*) OR TS=(drug NEAR/1 level*) OR TS=(serum NEAR/1 concentration*) OR TS=(plasma NEAR/1 concentration*) OR TS=(blood NEAR/1 concentration*) OR TS=(drug NEAR/1 concentration*) OR TS=(drug NEAR/1 monitor*) OR TS=(positron NEAR/1 emission NEAR/1 tomogra*) OR TS=(PET NEAR/1 scan*) OR TS=(single NEAR/1 photon NEAR/1 emission*) OR TS=SPECT OR TS=(CAT NEAR/1 Scan)) |
| Cochrane Library |
| ("aripiprazole" OR "aripiprazol*" OR "abilify" OR "opc14597") AND  ([mh "positron emission tomography"] OR [mh "Tomography, Emission-Computed, Single-Photon"] OR [mh "single photon emission computed tomography computed tomography"] OR (positron NEAR/1 emission NEAR/1 tomogra* ) OR (PET NEAR/1 scan*) OR (tomography, emission NEAR/1 computed, single NEAR/1 photon) OR (single NEAR/1 photon NEAR/1 emission*) OR SPECT OR (CAT NEAR/1 Scan) OR (single NEAR/1 photon NEAR/1 emission) OR (single NEAR/1 photon NEAR/1 emission NEAR/1 computed NEAR/1 tomography NEAR/1 computed NEAR/1 tomograph*):ti,ab,kw OR (drug NEAR/1 monitor*):ti,ab,kw OR (serum NEAR/1 level*) OR (plasma NEAR/1 level*) OR (blood NEAR/1 level*) OR (drug NEAR/1 level*) OR (serum NEAR/1 concentration*) OR (plasma NEAR/1 concentration*) OR (blood NEAR/1 concentration*) OR (drug NEAR/1 concentration*)):ti,ab,kw |
| PsycINFO |
| ("aripiprazole" OR "aripiprazol*" OR "abilify" OR "opc14597")  AND (MA "positron emission tomography" OR "positron emission tomogra*" OR "pet scan*" OR MA "tomography, emission computed, single photon" OR "single photon emission*" OR "SPECT" OR "CAT Scan" OR MA "single photon emission computed tomography computed tomography" OR MA "Drug Monitoring" OR "Drug Monitoring" OR "serum level*" OR "plasma level*" OR "blood level*" OR “drug level*" OR "serum concentration*" OR "plasma concentration*" OR "blood concentration*" OR “drug concentration*") NOT (MA "Animals" NOT MA "humans")  ***Table S2.*** ***Inclusion and exclusion criteria for study eligibility (PICOS).***   \|  \| Inclusion criteria \| Exclusion criteria \| \| --- \| --- \| --- \| \| Population \| - Psychiatric patients treated with aripiprazole^B^ - Indications for treatment is schizophrenia or related disorders or bipolar disorder^B^ \| - Non-human subjects - Healthy volunteers, non-psychiatric patients^B^ - Postmortem studies - Maternal use during pregnancy or lactation \| \| Intervention \| - Psychotropic monotherapy arm or period of observation (at least one blood level measurement before add-on therapy) - Treatment duration long enough to reach steady state (14 days)^C^ \| - Blood level is not measured in the steady state^C^ - Studies primarily comparing blood analysis techniques \| \| Outcome(s) \| - Drug concentrations measured in the blood (serum or plasma) - For concentration/effect studies: direct clinical outcome measures, i.e., safety or efficacy using a standardized rating scale (e.g., HAMD, MADRS, CGI)^A^ - For neuroimaging studies: dopamine D_2/3_ receptor occupancy \| - No mean or median blood level reported \| \| Study Design \| - observational and interventional studies are included - Reviews and meta-analyses investigating a concentration/ effect relationship for the relevant drug \| - Reviews and experts’ opinions - Gray literature - Case reports and case series \| \| Other \| - Reviews & meta-analysis investigating a concentration/effect relationship for ARI \| - Papers containing the same data - No abstract available - Data from simulation studies \|   ^A^ Biomarkers (e.g. QTc-time) are not regarded a direct clinical outcome measure.  ^B^ Not applicable for neuroimaging studies.  ^C^ Not applicable for neuroimaging studies and for studies, in which injectable formulations were administered. |

# ***Table S3. Detailed information on all included trials for oral formulations***

| **Author, year** | **Country** | **Design** | **Subjects** **(* = estimated from original data)** | **Mean Dose (range) [mg/day]** | **Mean ARI BL (range) [ng/ml]** | **Mean AM BL (range) [ng/ml]** | **Comment** |
| --- | --- | --- | --- | --- | --- | --- | --- |
| **Hwang et al., 2015** | Taiwan | Cluster RCT with fixed doses (15 mg) comparing fast vs. slow switching | N=79, SCZ or SD (clinically stable; PANNS <55), 40.5% Males, age 39.5 | 15 | 206±114* | NA | Higher ARI BL correlated with greater reduction in BARS on day 56. Conc. diff. between CYP2D6*2/*10 genotypes. |
| **Hoekstra 2021** | Norway | RCT, efficacy and side effects compared to olanzapine and amisulpride | N=51 (ARI), SCZ spectrum disorders, age 32.1 | 15.7 | NA | NA | Only in men, amisulpride superior to ARI (PANSS). Conc. reported in DDD. |
| **Lin et al., 2011** | Taiwan | Prospective CS with flexible doses (mean 15mg) | N=45, SCZ or SD , 42.2% Males, age 39.6±10.7 (19-59) | 14.2±6.3 | 208±136 | 296±188 | Higher ARI BL in responders (20% decrease in PANSS score, p .05). Higher baseline PANNS in responders. Response rate 62.2%. 225 ng/ml found as cut-off. Higher conc. in oriental (50%-100%) than in western patients |
| **Nemoto et al., 2012** | Japan | Prospective CS; PK after paroxetine add-on; fixed ARI doses (mean 14.6 mg) | N=14, SCZ, 40% Males, age 31.1±13.0 (20-67) | 14.6 | 189±149 | 273± 201 | CGI decreased with increasing ARI BL; Paroxetine increased ARI and AM conc. up to 1.7- and 1.4-fold (not D-ARI) |
| **Nemoto et al., 2014** | Japan | Prospective CS; PK after Paroxetine and escitalopram (ESC) add-on | N=13, SCZ or pervasive developmental disorders, 61.5% Males, age 34.5 | 17 | 514±156 | 774±231 | No change in DIEPS or CGI. Paroxetine increased Ari and AM conc. by 1.7- and 1.5-fold (not D-ARI). No effect of ESC. Conc. normalized to 24mg/d |
| **Nakamura et al., 2009** | Japan | Prospective CS with fixed doses (mean 22); PK after carbamazepine add-on | N=18, SCZ, 66.7% Males, age 35.8 ± 13.2 | 22 | 274±113 | 397±155 | Higher response and less neurological AEs with decreasing ARI BL; Carbamazepine add-on decreased ARI and D-ARI conc. by 64% and 68%. |
| **Citrome et al., 2007** | US | Prospective PK CS with carbamazepine add-on | N=6, SCZ or SD, 100% Males, age 39 (25–53) | 30 | 360±65 | 466 | BL ARI decreased after carbamazepin. No change in clinical effects |
| **Veselinovic et al., 2019** | Germany | Cohort nested in RCT; flexible design (mean 15.5); Comed. with other AP allowed | N=11, SCZ, 72.7% Males, age 34.9 | 15.5±6.0 | 205±196 | NA | Physical and mental well-being corr. neg. with estimated D2 receptor occupancy. No correlation with EPS scores. |
| **Findling et al., 2008** | US | Prospective CS | N=17, multiple psychiatric Dx, 66.7% Males, age 12.2 (10-17) | 25±4.3 | 385±122* | 509* | BL after 12h and after 24h did not vary much, no correlations for CGI changes and BL. No change in dyskinesia or akathisia symptoms. |
| **Zuo et al., 2006** | China (Han) | Prospective CS on ARI PK (12h) | N=12, SCZ, 50% Males, age 26.1±7.0 (18-45) | 10 | 388±109 | NA | Special dose regimen used in study. Baseline PANNS |
| **Kirschbaum et al., 2008** | Germany | CSS, TDM study on ARI response and side-effects | N=159 (SCZ) (67% Males, age 33.8±10.8 (19-66) | 19.9±8.12 | 216±139 | 292±172 | Low occurence of EPS (7%, comparable with placebo). IQR of responders: 124-286 ng/ml (AR) and 173-367 ng/ml (AM), IQR of patients with ADRs: 210- 335 ng/ml (ARI) and 245-375 ng/ml (AM). |
| **Egberts et al., 2020** | Germany and Austria | CSS with children and adolescents | N=130, 59.2% Males, multiple Dx, age 15 (7-19) | 15.1±5.4 (SCZ) | 243±138 (SCZ) | NA | Doses and conc. comparable to adult range. Conc. of patients with and without EPS did not differ. IQR of responders with SCZ: 106-375 ng/ml. |
| **Steen 2017** | Norway | Prospective CSS on cognitive function (TOP study); flexible doses, multiple Dx; N=373 | N=76 (SCZ), naturalistic, median age 28 | NA | NA | NA | Better attention/ working memory nominally associated with higher BL. |
| **Nagai 2012** | Japan | Prospective CS after fixed doses of ARI | N=70, 51.4% Males, SCZ, age 38.7±15.16 | 12.2 | 231±147 | 339±162 | Prolactin conc. not conc.- but sex-dependent |
| **Kim 2008** | Korea | Population model from prospective genotyping CS | N=80, 42,5% Males, SCZ, SD, schizophreniform disorder, age 35.2±9.8 | 24.3±6.7 | 446±221 | 566±229 | PK model for ARI and D-ARI to explain interindividual PK diff., CYP2D6 genotypes influenced ARI but no DARI BL |
| **Nakamura 2014** | Japan | Prospective CS on PK after haloperidol add-on | N=19, 47.4% Males,, SCZ, age 42.4±14.7 | 24 | 346±150 | 524±244 |  |
| **Nagai 2017** | Japan | Prospective CS on BL week 1 and 3 | N=26, 50% Males, SCZ, age 37.7±12.8 | 24 | 373 | 544 |  |
| **Bachmann et al., 2008** | Germany | Prospective CS in a naturalistic sample of adolescent patients | N=33, 54.5% Males, schizophrenia spectrum disorders, age 18.7±1.7 (13.5-21.6) | 12.9±6.4 | 142±123 | 194±125 | TDM in adolescents, no gender, age, smoking or sex-related PK diff. found. |
| **Castberg et al., 2007** | Norway | Prospective PK CS on effects of comedication in naturalistic setting | N=81, 56.8% Males, multiple Dx, age 34 (15-71) | 19.9 | 274± 185* | NA | Effect of CYP-interfering comed. discussed. No effect of gender or age |
| **Pozzi et al., 2016** | Italy | Retrospective CS | N=47, 59.6% Males, multiple Dx, age 15.5±2.4 | 10.8±6.6 | 187.0 ± 152 | NA | TDM in pediatric patients, Ct was close to the adult reference range |
| **Gründer 2008** | Germany | Prospective naturalistic cohort | N=128, 66% Males, SCZ or SD, mean age 33.8±10.7 | 20± 9 | 228 ± 142 | NA |  |
| **Van der Weide et al., 2015** | Netherlands | Retrospective CSS | N=130, multiple Dx | 17±7.6 A | 211±153A | 273±157* | CYP3A4*22 Polymorphism |
| **Jönsson et al., 2019** | Sweden | Retrospective TDM CSS with 12 antipsychotics | N=1610, 50% Males, multiple Dx, median age 33 (8-92) | 15.8±7.5 | 214±147 A* | 281±153 A* | PK diff in gender and age (cut-off 65 years) |
| **Jukic et al., 2019** | Norway | CSS CYP2D6 genotyping study | N=890, 48.7% Males, multiple Dx, age 37.8±16.8 | 14.5±7.7 | NA | NA | Increases in aripiprazole AM BL in poor and intermediate metabolizers (30% lower doses). No effect of genotype on treatment failure (AP switch) |
| **Eryilmaz et al., 2014** | Turkey | Retrospective CSS with valproate add-on after with electroconvulsive treatment (ECT) | N=69, 34.8% Males, BD, age 32.1±11.4 | 20 | 255±134 (121-389) | NA | AM BL after valproate coadministration changes by 23% |
| **Hendset et al., 2007** | Norway | Retrospective genotype (CYP 2D6) CSS, TDM database | N=62, 45.9% Males, multiple Dx, age 31.3 (17-84) | NA | NA | NA | CYP2D6 has a significant impact on ARI and AM BL of 4.3-fold and 4.2-fold. PMs need 30–40% lower doses |
| **Molden et al., 2006** | Norway | Retrospective PK CSS | N=118, 52.5% Males, multiple Dx, age 32.8 (15-63) | NA | NA | NA | PK (proportional dose-concentration relationship in population) |
| **Waade et al., 2009** | Norway | Retrospective CSS on comed. | N=223, 58% Males, multiple Dx, median age 31 (12-86) | NA | NA | NA | Effect of different comed. on C/D reported. CYP3A4 inducers resulted in 60% lower ARI exposure |
| **Jukic et al., 2021** | Norway | Retrospective CSS, genotyping | N=1265, 48% Males, multiple Dx, median age 34 (19-55) | 14.2±7.7 A* | 189±139 A* | 251 ±175 A* | Effect of CYP2D6 Genotypes on activity score |

# ***Table S4. Detailed information on all included trials for injectable formulations***

| **Author, year** | **Country** | **Design** | **Subjects** **(* = estimated from original data)** | **Mean Dose (range) [mg/day]** | **Oral Supplementation (except benzos and sleep medication)** | **Mean ARI BL (range) [ng/ml]** | **Mean AM BL (range) [ng/ml]** | **Comment** |
| --- | --- | --- | --- | --- | --- | --- | --- | --- |
| **Weiden et al., 2020 (same data as Risinger 2017 and Hard et al., 2017)** | USA | RCT, primary endpoint PK | N= 140, 73% Males, SCZ or SD, mean age 44.5±11.4 | 441 (q4wk), 882 (q6wk,) or 1064 (q8wk) **AL**,  1064 (q8wk) AL alternative formulation,  Multiple injections, gluteal | stabilized on oral AP, not ARI | C_avg_ 126, 131, 141 | NA | No change in ESRS and C-SSRS and ECG, population model calculated, maximum ARI BL were achieved 24.4–35.2 days after the last dose |
| **Hard 2018 et al., pop PK model** | USA | pop PK model from 4 studies, endpoint PK and safety, steady-state | N=343, 73% Males, SCZ or SD, mean age 45.2 ±10.8 | 441 (q4wk), 882 (q4wk,), 882 (q6wk,) or 1064 (q8wk) **AL**  Multiple injections | stabilized on oral AP, not ARI | C_max_ (median) 153 (114-203), 227 (165-311), 310 (227-447), 226 (167-333), 210 (148-292) | NA | Population model calculated, 1-day initiation regimen suitable for treatment with all doses |
| **Turncliff et al., 2014** | USA | RCT, Phase 1 pharmacokinetic study | N=43, 69.6% Males, SCZ or SD, mean age 42.5 | 221 and 441 **AL**  Single injection | stabilized on oral AP | C_max_ 57±22 and 46.8±23.6 | NA | No change in ESRS and C-SSRS and ECG, CYP 2D6 genotyping |
| **Hard et al., 2018** | USA | RCT primary endpoint PK | N = 133, 73.3 % Males, SCZ or SD, mean age 44.0 | 441 or 882 (1-day initiation regimen **AL nanocrystal dispersion gluteal** or 21-day initiation regimen **AL gluteal and deltoid**)  Single injection | stabilized on oral AP | C_28_ 441mg (1day): 183 ,441mg (21 day): 65, 882mg (1 day) 178, 882mg (21d) 99 | NA | SSRS, CGI-S remained stable, CYP2D6 poor metabolizers excluded |
| **Hard et al., 2019** | USA | RCT primary endpoint PK | n = 47, 72.3% Males, SCZ or SD, mean age 48.6 | 441, **AL nanocrystal dispersion**  Single injection, deltoid and gluteal | stabilized on oral AP | C_28_ 124 (C_max_ 185)* | C_max_ 252.8 | SAS total score >3 one patient, no akathisia or dyskinesia (AIMS, BARS, CSSRS), deltoid vs. gluteal, CYP2D6 poor metabolizers excluded |
| **Raoufinia et al., 2017 – study 2** | USA | RCT, primary endpoint PK | N=86, SCZ, mean age 43.9 | 400 (q4wk) **AM**  Multiple injections (five), 4/5 deltoid | stabilized on oral AP | C_min_ 239±133 | C_min_ 320.2±168.9 | SAS, AIMS, BARS, CSSRS remained stable, deltoid vs. gluteal |
| **Mallikaarjun et al., 2013** | USA | RCT, 25 weeks, show consistency of plasma levels with reference range for oral dosing | N = 47, 72.3% Males, SCZ, mean age 45.2 | 200, 300, 400 (q4wk) **AM**  Multiple injections (five), deltoid and gluteal | ARI Days 1-14, no other | C_min_ 95±86, 156±68, 212±113  Cmean 68.9, 175, 190 | NA | PANSS, CGI-S, SAS, AIMS, BARS remained stable, deltoid vs. gluteal |
| **Mauri et al., 2020** | Italy | Prospective observational CS over 12 months in BD patients | N= 56, 20% Males, mean age 41.9, **BD** with manic predominance | 300 or 400 (q4wk) mean 358 mg ± 88 **AM**  Multiple injections, deltoid or gluteal according to patients preference | No AP | C_min_ range 113 -132 | NA | BPRS, HAMD, PANSS, and MRS showed initial reduction. Relation between intraindividual variation of ARI BLs and variation of BPRS. Limit of <150 ng/ml suggested for LAI in BP for depressive symptoms and positive symptoms. |
| **Raoufinia et al., 2017 – study 1** | USA | RCT, primary endpoint PK | N=33, SCZ, mean age 44.5 | 400 **AM**  Single injection, deltoid and gluteal | stabilized on oral AP | C_28_ 94.4 | C_28_ 21.4 n=33* | SAS, AIMS, BARS, CSSRS remained stable |
| **Potkin et al., 2013** | USA | Prospective CS | N = 60, 83% Males, SCZ, mean age 43.2 | 400, **AM**  Single injection | stabilized on oral AP | C_28_: 93±42 | NA | PANSS, CGI-S, SAS, AIMS, BARS, CSSRS remained stable |
| **De Filippis et al., 2013** | Italy | Prospective CS, i.m. in acute phase of agitation | N = 7, BD and SCZ with **acute** phase of agitation | 9.75  Single injection | No AP, except benzos | 87.2 | NA | clinical response poorly correlated with BL |
| **Boulton et al., 2008 Study 2** | USA | Prospective PK study for **acute** effects after i.m. for 4 days | N= 31, 81% Males, SCZ, mean age 39 | 1-30  Four injections | No oral AP or ARI | NA | NA | linear PK profile |

*from diagram using webplotdigitizer

# ***Table S5. PET studies reporting D2 receptor occupancy and aripiprazole (ARI) blood concentrations***

| **Author, year** | **PET tracer** | **Design** | **Subjects** | **Exclusion criteria** | **Mean Dose (range) [mg/day]** | **Mean ARI BL (range) [ng/ml]** | **Mean receptor occupancy (%)** | **EC_50_ [ng/ml]** | **EC_90_ (estimated from EC_50_) [ng/ml]** | **Comment** |
| --- | --- | --- | --- | --- | --- | --- | --- | --- | --- | --- |
| Yokoi et al., 2002 | [^11^C]raclopride | Cohort study, dose response PET scans of fixed doses of ARI taken for 14 days, trough samples analyzed by HPLC/ UV | N=15; healthy volunteers; age 32±9; 100% males | NA | 10±12.8 (0.5-30) | NA (only in diagram) | D_2/3_: 66.8±25.0 (c); 66.9±21.59 (p) | NA | NA | Hyberbolic relation between peak ARI conc. and D_2_ occup. (p) |
| Mamo et al., 2007; Mizrahi et al., 2009 (same cohort) | [^11^C]raclopride, [18F]setoperone, [^11^C]WAY100635 | RCT, 3 PET scans after ARI taken for 14 days; diagnosis acc. to DSM-4. Peak levels measured with LC/MS, clinical efficacy assessments | N=12; SCZ or SD; age 31±7; 75% males | Substance abuse, depot APs in last 6 months; change in or other psychotropic medications | 18.8±7.7 (10-30) | 221±179 | D_2/3_: 86.6±3.7 (p), 92.9±5.7 (c), 91.0±4.0 (cs); 5-HT_2_: 54.0±15.3 (tc), 59.4±12.9 (fc); 5-HT_1A_: 16.2±14.3 (tc), 16.5±13.8 (fc) | NA | NA | ARI and DARI conc. correlated with D_2_ occup. (p and s). No corr. between occup. and clinical or well-being scores. |
| Gründer et al., 2008 | [^18^F]fallypride | Cohort study with medication-free vs. medicated patients, trough serum concentrations in steady-state measured with HPLC | N=16/8 (medicated/ unmedicated); SCZ or SD (DSM-4); age 30; 94% males | Psychotropic medication for at least 6 months | 18.8±7.2 (5-30) | 245±307 | D_2/3_: 83±1 (p), 84±1 (c), 85±7 (t) | 10±4 (p)  9±4 (c) | 90 (p), 81 (c) | Complete occup. with ARI conc. > 100–150 ng/ml. Lower EC_50_ in thalamus (6±2 ng/ml) |
| Kegeles et al., 2008 | [^18^F]fallypride | Cohort study, fixed doses, serum conc. measured with RP LC/UV | N=19; SCZ or SD (DSM-4); age 29; 79% males | Substance abuse, psychotropic medication for last 21 days | 13.9±11 (2-40) | NA (excl. in analysis) | D_2/3_: NA  79.8±14.8 (s) in 15 mg | ED_80_ 5.63±1.0 (s) ~ 100 ng/ml | NA | Dose correlated with ARI conc., PANSS positive scale corr. with D_2_ occup. (s). No EPS. |
| Ito et al., 2012 | [^11^C]raclopride, L-[ß-^11^C]DOPA | Cohort study, PET scans after single dose of ARI, serum conc. measured with LC/MS | N=12; healthy volunteers; age 24.1±3.2; 100% males | Somatic, neurological and psychiatric disorders, drug abuse | 5.3±2.3 (3-9) | 23.8±11.3 | D_2/3_: 67.2±9.7(c), 64.3±8.9 (p) | NA | NA | No changes in dopamine synthesis capacity. |
| Kim et al., 2012 | [^11^C]raclopride | RCT, single dose of aripiprazole after fasting, sampling up to 120h | N=18; healthy volunteers; age 22.9±2.4; 100% males | Somatic and psychiatric disorders; caffeine, grapefruit, alcohol, and smoking | 12.7±11.5 (2-30) | Peak: 3.4±0.9 per mg | D_2/3_: 61.7±21.2 (s) | 11.1 (s) | 99.9 (s) | Values reported for PK model; PK/PD model estimates EC_90_ of 77.4 ng/ml (s) |
| Takahata et al., 2012 | [^11^C]raclopride, [11C]FLB457 | Cohort single dose study on extrastriatal binding of ARI, peak conc. measured with LC/MS | N=11; healthy volunteers; age 23.7±4.0; 100% males | Psychiatric or neurologic disorders, substance abuse | 6 | 29.4±4.8 | D_2/3_: 74.1±6.7 (c), 70.1±6.3 (p), 57.6±6.7 (t), 51.3±9.2 (fc), 58.4±3.0 (tc) | 9.9 (s), 12.2 (p), 18.9 (t), 24.3 (fc), 18.2 (tc) | 89.1 (s), 109.8 (p) | Concentration reported for raclopride scans; lower in FLB457. No preferential extrastriatal binding of ARI |
| Kim et al., 2013 | [^11^C]raclopride and [^18^F]FDG | RCT, PET and fMRI study with single dose of aripiprazole after fasting, sampling before scans | N=15; healthy volunteers; age 23.1±2.4; 100% males | Somatic and psychiatric disorders; caffeine, grapefruit, alcohol, and smoking | 12.4±11.4 (2-30) | 15.0±14.3 | D_2/3_: 50.2± 22.0 (s) | NA | NA | Reaction times in working memory task and metabolic change in frontal lobe pos. corr. with D_2_ occup. |
| Shin et al., 2018 | [^11^C]raclopride | Cohort study; PET and fMRI scans performed after flexible ARI; trough samples in the steady-state | N=7; SCZ (DSM-4); age 32; 28.6% males | Comedication; caffeine, grapefruit, alcohol, and smoking | 14.2±12 (2-30) | 290±325 | D_2/3_: 65.0±8.6 (s) | NA | NA | Error rates and reaction time in working memory task pos. corr. with D_2_ occup. |

| Studies using oral aripiprazole | | | | | | | | | | | |
| --- | --- | --- | --- | --- | --- | --- | --- | --- | --- | --- | --- |
| No | Reference | Q1  *Patient sample* | Q2  Diagnosis | Q3  Comedication | Q4  Dose design | | Q5  Analytical method | | Q6  Sampling | Q7  Design | Score |
| 1 | Hwang et al., 2015 [1] | o | xo | x | x | | x | | xx | xo | 7/10 |
| 2 | Hoekstra et al., 2021 [2] | x | xo | o | o | | ? | | ?? | ?x | 3/10 |
| 3 | Lin et al., 2011 [3] | o | ?o | x | o | | x | | xx | xx | 6/10 |
| 4 | Nemoto et al., 2012 [4] | o | xx | o | x | | x | | xx | xx | 8/10 |
| 5 | Nemoto et al., 2014 [5] | o | xo | o | x | | x | | xx | xx | 7/10 |
| 6 | Nakamura et al., 2009 [6] | o | xx | o | x | | x | | xx | xx | 8/10 |
| 7 | Citrome et al., 2007 [7] | o | xo | x | x | | x | | xx | xx | 8/10 |
| 8 | Veselinovic et al., 2019 [8] | x | xx | o | o | | x | | xx | xx | 8/10 |
| 9 | Findling et al., 2008 [9] | o | ?o | x | x | | x | | xx | xx | 7/10 |
| 10 | Zuo et al., 2006 [10] | o | xx | x | x | | x | | xx | xx | 9/10 |
| 11 | Kirschbaum et al., 2008 [11] | x | xx | o | o | | x | | xx | xx | 8/10 |
| 12 | Egberts et al., 2020 [12] | o | xx | o | x | | x | | xx | ox | 7/10 |
| 13 | Steen et al., 2017 [13] | x | xo | o | o | | x | | ox | ox | 5/10 |
| 14 | Nagai et al., 2012 [14] | o | xx | x | x | | x | | xx | ox | 8/10 |
| 15 | Kim 2008 [15] | o | ?o | x | o | | x | | ox | ox | 4/10 |
| 16 | Nakamura et al., 2014 [16] | o | xx | x | x | | x | | xx | ox | 8/10 |
| 17 | Nagai et al., 2017 [17] | o | xx | x | x | | x | | xx | xx | 9/10 |
| 18 | Bachmann et al., 2008 [18] | o | xo | x | o | | x | | xx | ox | 6/10 |
| 19 | Castberg et al., 2007 [19] | x | ?o | x | o | | x | | oo | ox | 4/10 |
| 20 | Pozzi et al., 2016 [20] | o | xo | o | o | | x | | xx | ox | 5/10 |
| 21 | Gründer 2008 et al., "TDM sample" [21] | x | ?o | ? | o | | ? | | x? | ?x | 3/10 |
| 22 | Van der Weide et al., 2015 [22] | o | ?o | o | o | | x | | xx | ox | 4/10 |
| 23 | Jönsson et al., 2019 [23] | x | ?o | o | o | | x | | xx | xx | 6/10 |
| 24 | Jukic et al., 2019 [24] | o | ?o | x | o | | x | | xx | ox | 5/10 |
| 25 | Eryilmaz et al., 2014 [25] | x | xx | o | x | | ? | | ox | xx | 7/10 |
| 26 | Hendset et al., 2007 [26] | o | ?o | x | o | | x | | xx | ox | 5/10 |
| 27 | Molden et al., 2006 [27] | x | ?o | x | o | | x | | oo | ox | 4/10 |
| 28 | Waade et al., 2009 [28] | x | ?o | x | o | | x | | xx | x? | 6/10 |
| 29 | Jukic et al., 2021 [29] | o | ?o | o | o | | x | | ox | ox | 3/10 |
| Studies using injectable formulations of aripiprazole | | | | | | | | | | | |
| No | Reference | Q1 | Q2 | Q3 | Q4 | | Q5 | | Q6 | Q7 | Score |
| 30 | Turncliff et al., 2014 [30] | o | xo | o | o | | x | | ox | xo | 4/10 |
| 31 | Raoufinia et al., 2017 single-dose [31] | o | xx | o | o | | x | | ox | xo | 5/10 |
| 32 | Raoufinia et al., 2017 multiple-dose [31] | o | xx | o | x | | x | | xx | xx | 8/10 |
| 33 | Weiden et al., 2020 [32] / Risinger et al., 2017 [33] Hard et al., 2017 [34] | o | xo | o | x | | ? | | xo | xx | 5/10 |
| 34 | Hard et al., 2018 pop PK model [35] | o | ?o | x | o | | x | | xx | x? | 5/10 |
| 35 | Hard et al., 2019 [36] | o | xo | o | o | | ? | | ox | xx | 4/10 |
| 36 | Mallikaarjun et al., 2013 [37] | o | xx | x | x | | ? | | xx | xx | 8/10 |
| 37 | Potkin et al., 2013 [38] | o | xx | o | o | | ? | | oo | xo | 3/10 |
| 38 | De Filippis et al., 2013 [39] | x | xo | o | x | | x | | ox | xo | 6/10 |
| 39 | Mauri et al., 2020 [40] | x | xx | x | o | | x | | xx | xo | 8/10 |
| 40 | Boulton et al., 2008 Study 2 [41] | o | xx | o | x | | x | | ox | xx | 7/10 |
| Dopamine receptor occupancy studies | | | | | | | | | | | |
| No | Reference | Q1 | Q2 | Q3 | | Q4 | | Q5 | Q6 | Q7 | Score |
| 41 | Mamo et al., 2007 [42], Mizrahi et al., 2009 [43] | x | xo | ? | | x | | ? | xx | xo | 6/10 |
| 42 | Kim et al., 2012 [44] | oH | xx | x | | o | | ? | ox | xo | 5/10 |
| 43 | Kim et al., 2013 [45] | oH | xx | x | | o | | ? | ox | oo | 4/10 |
| 44 | Yokoi et al., 2002 [46] | oH | ox | ? | | x | | ? | xx | xx | 6/10 |
| 45 | Gründer et al., 2008 (PET cohort) [21] | x | xo | ? | | o | | x | xx | xx | 7/10 |
| 46 | Kegeles et al., 2008 [47] | o | xo | ? | | x | | ? | xo | xx | 5/10 |
| 47 | Ito et al., 2012 [48] | oH | ?x | ? | | o | | ? | ox | xo | 2/10 |
| 48 | Takahata et al., 2012 [49] | o | ?x | ? | | o | | ? | ox | xo | 2/10 |
| 49 | Shin et al., 2018 [50] | o | xx | x | | o | | ? | xx | xx | 7/10 |

***Table S6. Rating result of general quality criteria for the therapeutic drug monitoring component for all studies***

# ***Table S7. Study type specific quality assessment - Cross-sectional studies***

| No | Study | Selection (Max 4 p): | | | | Comparability (Max 2 p): | Outcome (Max 3 p) | | Total score (x/8) |
| --- | --- | --- | --- | --- | --- | --- | --- | --- | --- |
|  |  | Q1 | Q2 | Q3 | Q4 | Q5 | Q6 | Q7 |  |
| 1 | Kirschbaum et al., 2008 | x | o | x | ? | xo | o | x | 4/8 |
| 2 | Egberts et al., 2020 | o | o | x | o | oo | o | x | 2/8 |
| 3 | Steen 2017 | x | o | x | x | ox | x | x | 6/8 |
| 4 | Van der Weide et al., 2015 | o | o | x | o | xx | x | x | 5/8 |
| 5 | Jönsson et al., 2019 | x | o | x | o | xx | x | x | 6/8 |
| 6 | Jukic et al., 2019 | o | o | o | x | xx | x | x | 5/8 |
| 7 | Eryilmaz et al., 2014 | x | o | o | o | xo | x | x | 4/8 |
| 8 | Hendset et al., 2007 | o | o | x | o | xx | x | x | 5/8 |
| 9 | Molden et al., 2006 | x | o | x | o | xx | x | x | 6/8 |
| 10 | Waade et al., 2009 | x | o | x | o | xx | x | x | 6/8 |
| 11 | Jukic et al., 2021 | o | o | x | o | xx | x | x | 5/8 |

# ***Table S8. Study type specific quality assessment – Cohort studies***

| No | Study | Selection (Max. 4 p): | | | | Comparability (Max 2p) | Outcome (Maximum 3 p) | | | | Total score (x/10) |
| --- | --- | --- | --- | --- | --- | --- | --- | --- | --- | --- | --- |
|  |  | Q1 | Q2 | Q3 | Q4 | Q5 | Q6 | Q7 | Q8 | Q9 |  |
| 1 | Lin et al., 2011 | o | o | ? | x | ox | ? | x | o | x | 4/10 |
| 2 | Nemoto et al., 2012 | o | o | x | x | xx | ? | x | x | o | 6/10 |
| 3 | Nemoto et al., 2014 | o | o | x | x | xx | ? | x | x | x | 7/10 |
| 4 | Nakamura et al., 2009 | o | o | ? | x | xx | ? | o | x | o | 4/10 |
| 5 | Citrome et al., 2007 | o | x | ? | x | ox | x | x | o | o | 5/10 |
| 6 | Veselinovic et al., 2019 | o | x | x | x | ox | o | x | o | x | 6/10 |
| 7 | Findling et al., 2008 | o | x | ? | x | xx | ? | x | x | o | 6/10 |
| 8 | Zuo 2006 | o | o | ? | x | xx | x | x | x | o | 6/10 |
| 9 | Nagai 2012 | o | o | ? | o | xx | x | x | x | x | 6/10 |
| 10 | Kim 2008 | o | o | ? | o | xx | x | x | ? | x | 5/10 |
| 11 | Nakamura 2014 | o | x | ? | ? | xx | x | x | x | x | 7/10 |
| 12 | Nagai 2017 | o | o | x | x | xx | x | x | x | x | 8/10 |
| 13 | Bachmann et al., 2008 | o | o | o | o | xx | x | x | x | x | 6/10 |
| 14 | Castberg et al., 2007 | x | o | o | o | xx | x | o | x | o | 5/10 |
| 15 | Pozzi et al., 2016 | o | o | o | o | xx | x | x | x | x | 6/10 |
| 16 | Gründer 2008 "Clinical sample" | x | o | ? | ? | oo | x | x | ? | x | 4/10 |
| 17 | Potkin 2013 | o | x | x | x | xx | o | x | x | o | 7/10 |
| 18 | De Filippis 2013 | o | o | x | x | xo | o | x | x | x | 6/10 |
| 19 | Mauri 2020 | x | o | x | x | oo | x | x | o | x | 6/10 |
| 20 | Boulton 2008 Study 2 | o | x | x | x | xx | x | o | x | x | 8/10 |
| 21 | Hard 2017 | o | x | x | x | oo | x | x | x | x | 7/10 |
| 22 | Hard 2018 pop PK model* | o | o | x | x | ox | x | x | ? | x | 6/10 |
| 23 | Yokoi 2002 | oH | x | ? | x | xo | x | x | x | o | 6/10 |
| 24 | Gründer 2008 (PET cohort) | x | x | ? | o | xx | x | x | x | x | 8/10 |
| 25 | Kegeles 2008 | o | o | ? | x | xx | o | x | o | o | 4/10 |
| 26 | Ito 2012 | oH | x | ? | x | xx | x | x | x | x | 8/10 |
| 27 | Takahata 2012 | o | o | ? | x | xx | x | x | x | x | 7/10 |
| 28 | Shin 2018 | o | o | ? | o | xx | x | x | x | o | 5/10 |

*Quality assessment was performed for each study separately; lowest rating was used for the overall rating.

***Figure S9. Study type specific quality assessment – Randomized controlled trials***


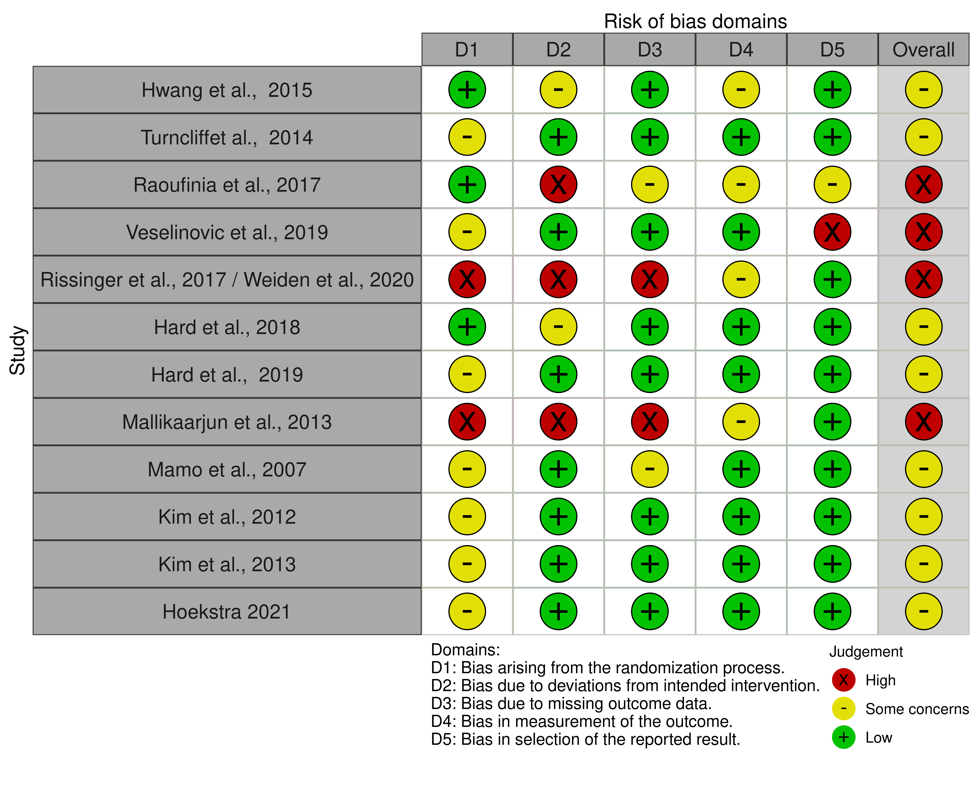


# ***Figure S10. Risk for Bias in randomized controlled trials***

***
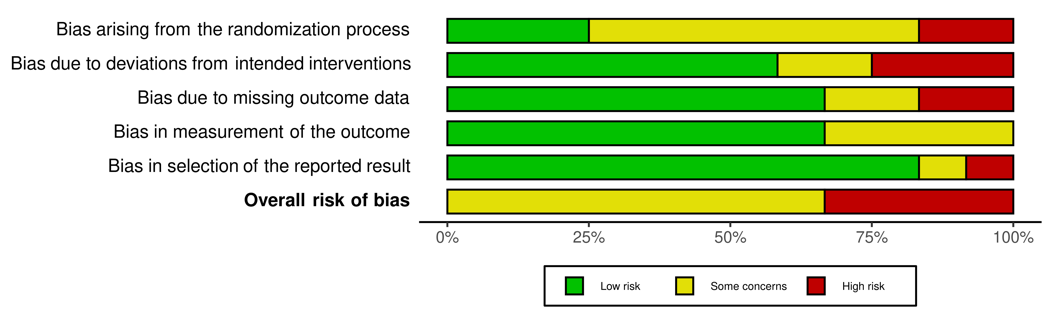
***

***Figure S11. Quality assessment results for TDM component (dark red, unclear; red insufficient; green, sufficient)***

**
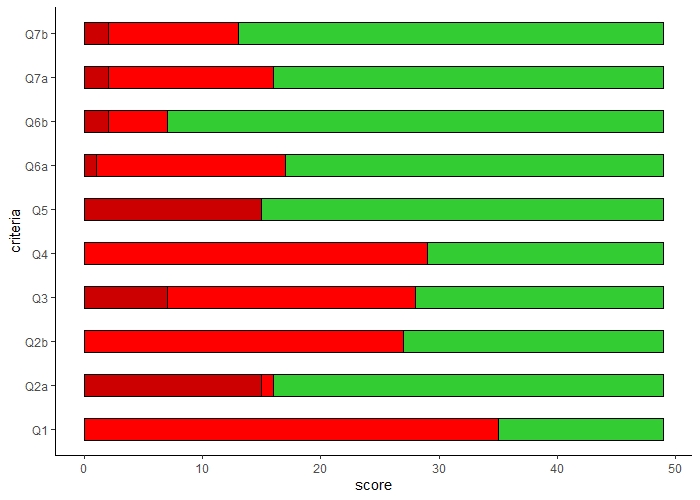
**

***Figure S12. Mean Aripiprazole Dose [mg/day] Versus Mean Active Moiety blood concentration [ng/ml] (β-coefficient = 18.030 (6.491-29.569), r = 0.787, P .007, y= 12.065 + 18.030 * x).***

**
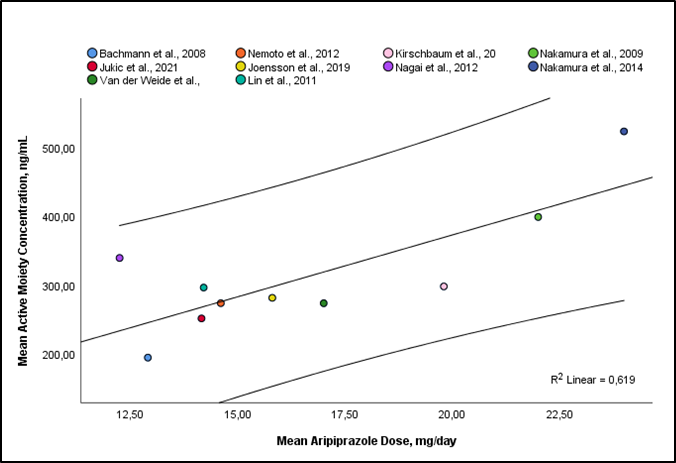
**

***Figure S13. Overall mean AM (ARI+D-ARI) concentration estimate, N=3,332 (corresp. mean ARI conc.: 218.11 ng/ml)***

***
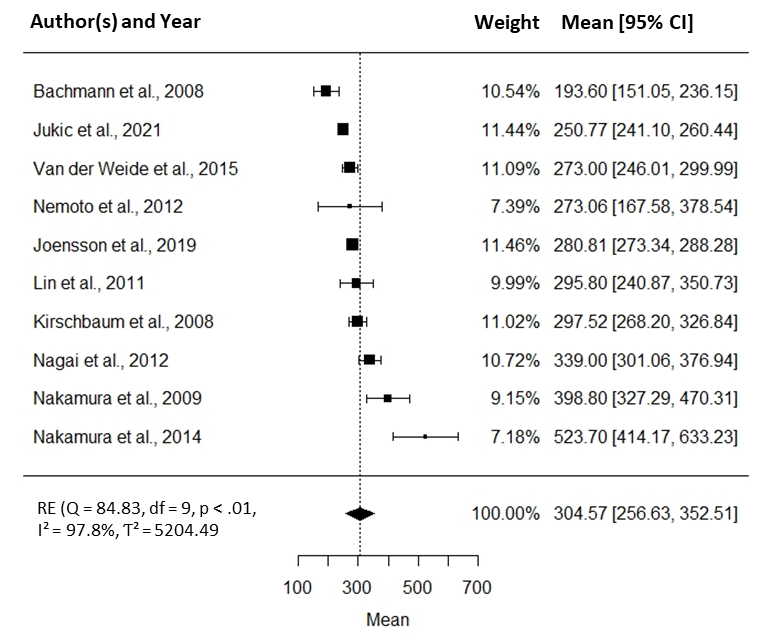
***

***Table S14. Subgroup analysis of potential moderators on mean ARI concentration (*significant result p< .05)***

| **Criterion (N studies)** | **Chi^2^** | **df** | **p** | **I^2^** |
| --- | --- | --- | --- | --- |
| Dose design [Fixed vs. flexible] | 5.01 | 1 | 0.025* | 93.94% |
| Trough level [Yes vs. No] | 0.07 | 1 | 0.798 | 93.32% |
| Ethnic group Caucasian [Yes vs. No] | 2.37 | 1 | 0.124 | 94.67% |
| Diagnosis Schizophrenia [Yes vs. No] | 0.74 | 1 | 0.388 | 95.26% |

**S15 Phenotype classifications**

Dependent upon their pattern in CYP2D6 allele variants, studies usually classified patients according to their expected phenotype. For CYP2D6 there is no uniformly agreed way in which to relate genotype to phenotype. In addition, CYP2D6 allele variants that have been used to classify genotypes widely differ among studies. CYP2D6*1 and *2 (allele A in rs16947 or allele C in rs1135840) allele have been shown to have increased enzyme activity (EM), referring to the “normal” wild type (wt alleles). However, metabolic ratio between *1 and *2 may differ. The phenotype of CYP2D6 poor metabolizer is caused mainly by the mutant alleles (vt) *3, *4, *5, *6, *9 which are associated with an enzymatic loss of function. The alleles CYP2D6*3, *4, and *5 are mostly found in Caucasians and have been shown to be rare in Asians. CYP2D6*14 seems more specific for poor metabolizing activity in Asian populations [51]. The vt alelles *10 and *41 are associated with IMs due to a decreased/unstable enzyme activity. Generally, IMs are thought to possess at least one decreased activity allele. Classifications vary widely. Patients with a gene duplication (*1xN, *2xN) in absence of any of the determined mutations or sometimes when there are multiple copies of an allele are usually classified as UM with an increased activity.

**S16. Full list of included studies/ publications**

1. Hwang TJ, Lo WM, Chan HY, Lin CF, Hsieh MH, Liu CC, et al. Fast Versus Slow Strategy of Switching Patients With Schizophrenia to Aripiprazole From Other Antipsychotics. Journal of clinical psychopharmacology. 2015;35(6):635‐44.

2. Hoekstra S, Bartz-Johannessen C, Sinkeviciute I, Reitan SK, Kroken RA, Løberg EM, et al. Sex differences in antipsychotic efficacy and side effects in schizophrenia spectrum disorder: results from the BeSt InTro study. NPJ Schizophr. 2021;7(1):39.

3. Lin SK, Chen CK, Liu YL. Aripiprazole and dehydroaripiprazole plasma concentrations and clinical responses in patients with schizophrenia. J Clin Psychopharmacol. 2011;31(6):758-62.

4. Nemoto K, Mihara K, Nakamura A, Nagai G, Kagawa S, Suzuki T, et al. Effects of paroxetine on plasma concentrations of aripiprazole and its active metabolite, dehydroaripiprazole, in Japanese patients with schizophrenia. Therapeutic drug monitoring. 2012;34(2):188‐92.

5. Nemoto K, Mihara K, Nakamura A, Nagai G, Kagawa S, Suzuki T, et al. Effects of escitalopram on plasma concentrations of aripiprazole and its active metabolite, dehydroaripiprazole, in Japanese patients. Pharmacopsychiatry. 2014;47(3):101‐4.

6. Nakamura A, Mihara K, Nagai G, Suzuki T, Kondo T. Pharmacokinetic and pharmacodynamic interactions between carbamazepine and aripiprazole in patients with schizophrenia. Ther Drug Monit. 2009;31(5):575-8.

7. Citrome L, MacHer JP, Salazar DE, Mallikaarjun S, Boulton DW. Pharmacokinetics of aripiprazole and concomitant carbamazepine. Journal of clinical psychopharmacology. 2007;27(3):279‐83.

8. Veselinović T, Scharpenberg M, Heinze M, Cordes J, Mühlbauer B, Juckel G, et al. Dopamine D2 Receptor Occupancy Estimated From Plasma Concentrations of Four Different Antipsychotics and the Subjective Experience of Physical and Mental Well-Being in Schizophrenia: results From the Randomized NeSSy Trial. Journal of clinical psychopharmacology. 2019;39(6):550‐60.

9. Findling RL, Kauffman RE, Sallee FR, Carson WH, Nyilas M, Mallikaarjun S, et al. Tolerability and pharmacokinetics of aripiprazole in children and adolescents with psychiatric disorders: an open-label, dose-escalation study. J Clin Psychopharmacol. 2008;28(4):441-6.

10. Zuo XC, Liu SK, Yi ZY, Xie ZH, Li HD. Steady-state pharmacokinetic properties of aripiprazole 10 mg PO g12h in Han Chinese adults with schizophrenia: A prospective, open-label, pilot study. Current therapeutic research, clinical and experimental. 2006;67(4):258-69.

11. Kirschbaum KM, Müller MJ, Malevani J, Mobascher A, Burchardt C, Piel M, et al. Serum levels of aripiprazole and dehydroaripiprazole, clinical response and side effects. The world journal of biological psychiatry : the official journal of the World Federation of Societies of Biological Psychiatry. 2008;9(3):212-8.

12. Egberts K, Reuter-Dang SY, Fekete S, Kulpok C, Mehler-Wex C, Wewetzer C, et al. Therapeutic drug monitoring of children and adolescents treated with aripiprazole: observational results from routine patient care. J Neural Transm (Vienna). 2020.

13. Steen NE, Aas M, Simonsen C, Dieset I, Tesli M, Nerhus M, et al. Serum levels of second-generation antipsychotics are associated with cognitive function in psychotic disorders. The world journal of biological psychiatry : the official journal of the World Federation of Societies of Biological Psychiatry. 2017;18(6):471-82.

14. Nagai G, Mihara K, Nakamura A, Suzuki T, Nemoto K, Kagawa S, et al. Prolactin concentrations during aripiprazole treatment in relation to sex, plasma drugs concentrations and genetic polymorphisms of dopamine D2 receptor and cytochrome P450 2D6 in Japanese patients with schizophrenia. Psychiatry and clinical neurosciences. 2012;66(6):518-24.

15. Kim JR, Seo HB, Cho JY, Kang DH, Kim YK, Bahk WM, et al. Population pharmacokinetic modelling of aripiprazole and its active metabolite, dehydroaripiprazole, in psychiatric patients. British journal of clinical pharmacology. 2008;66(6):802-10.

16. Nakamura A, Mihara K, Nemoto K, Nagai G, Kagawa S, Suzuki T, et al. Lack of correlation between the steady-state plasma concentrations of aripiprazole and haloperidol in Japanese patients with schizophrenia. Ther Drug Monit. 2014;36(6):815-8.

17. Nagai G, Mihara K, Nakamura A, Nemoto K, Kagawa S, Suzuki T, et al. Prediction of an Optimal Dose of Aripiprazole in the Treatment of Schizophrenia From Plasma Concentrations of Aripiprazole Plus Its Active Metabolite Dehydroaripiprazole at Week 1. Ther Drug Monit. 2017;39(1):62-5.

18. Bachmann CJ, Rieger-Gies A, Heinzel-Gutenbrunner M, Hiemke C, Remschmidt H, Theisen FM. Large variability of aripiprazole and dehydroaripiprazole serum concentrations in adolescent patients with schizophrenia. Ther Drug Monit. 2008;30(4):462-6.

19. Castberg I, Spigset O. Effects of comedication on the serum levels of aripiprazole: evidence from a routine therapeutic drug monitoring service. Pharmacopsychiatry. 2007;40(3):107-10.

20. Pozzi M, Cattaneo D, Baldelli S, Fucile S, Capuano A, Bravaccio C, et al. Therapeutic drug monitoring of second-generation antipsychotics in pediatric patients: an observational study in real-life settings. European journal of clinical pharmacology. 2016;72(3):285-93.

21. Grunder G, Fellows C, Janouschek H, Veselinovic T, Boy C, Brocheler A, et al. Brain and plasma pharmacokinetics of aripiprazole in patients with schizophrenia: An F-18 fallypride PET study. American Journal of Psychiatry. 2008;165(8):988-95.

22. van der Weide K, van der Weide J. The Influence of the CYP3A4*22 Polymorphism and CYP2D6 Polymorphisms on Serum Concentrations of Aripiprazole, Haloperidol, Pimozide, and Risperidone in Psychiatric Patients. J Clin Psychopharmacol. 2015;35(3):228-36.

23. Jönsson AK, Spigset O, Reis M. A Compilation of Serum Concentrations of 12 Antipsychotic Drugs in a Therapeutic Drug Monitoring Setting. Ther Drug Monit. 2019;41(3):348-56.

24. Jukic MM, Smith RL, Haslemo T, Molden E, Ingelman-Sundberg M. Effect of CYP2D6 genotype on exposure and efficacy of risperidone and aripiprazole: a retrospective, cohort study. The lancet Psychiatry. 2019;6(5):418-26.

25. Eryilmaz G, Hizli Sayar G, Özten E, Gül IG, Karamustafalioğlu O, Yorbik Ö. Effect of valproate on the plasma concentrations of aripiprazole in bipolar patients. International journal of psychiatry in clinical practice. 2014;18(4):288-92.

26. Hendset M, Hermann M, Lunde H, Refsum H, Molden E. Impact of the CYP2D6 genotype on steady-state serum concentrations of aripiprazole and dehydroaripiprazole. European journal of clinical pharmacology. 2007;63(12):1147-51.

27. Molden E, Lunde H, Lunder N, Refsum H. Pharmacokinetic variability of aripiprazole and the active metabolite dehydroaripiprazole in psychiatric patients. Ther Drug Monit. 2006;28(6):744-9.

28. Waade RB, Christensen H, Rudberg I, Refsum H, Hermann M. Influence of comedication on serum concentrations of aripiprazole and dehydroaripiprazole. Ther Drug Monit. 2009;31(2):233-8.

29. Jukić MM, Smith RL, Molden E, Ingelman-Sundberg M. Evaluation of the CYP2D6 Haplotype Activity Scores Based on Metabolic Ratios of 4,700 Patients Treated With Three Different CYP2D6 Substrates. Clinical pharmacology and therapeutics. 2021;110(3):750-8.

30. Turncliff R, Hard M, Du Y, Risinger R, Ehrich EW. Relative bioavailability and safety of aripiprazole lauroxil, a novel once-monthly, long-acting injectable atypical antipsychotic, following deltoid and gluteal administration in adult subjects with schizophrenia. Schizophr Res. 2014;159(2-3):404-10.

31. Raoufinia A, Peters-Strickland T, Nylander A-G, Baker RA, Eramo A, Jin N, et al. Aripiprazole once-monthly 400 mg: Comparison of pharmacokinetics, tolerability, and safety of deltoid versus gluteal administration. International Journal of Neuropsychopharmacology. 2017;20(4):295-304.

32. Weiden PJ, Du Y, von Moltke L, Wehr A, Hard M, Marandi M, et al. Pharmacokinetics, Safety, and Tolerability of a 2-Month Dose Interval Regimen of the Long-Acting Injectable Antipsychotic Aripiprazole Lauroxil: Results From a 44-Week Phase I Study. CNS Drugs. 2020;34(9):961-72.

33. Risinger R, Hard M, Weiden PJ. A Phase-1 Study Comparing Pharmacokinetic and Safety Profiles of Three Different Dose Intervals of Aripiprazole Lauroxil. Psychopharmacol Bull. 2017;47(3):26-34.

34. Hard ML, Mills RJ, Sadler BM, Wehr AY, Weiden PJ, von Moltke L. Pharmacokinetic Profile of a 2-Month Dose Regimen of Aripiprazole Lauroxil: A Phase I Study and a Population Pharmacokinetic Model. Cns Drugs. 2017;31(7):617-24.

35. Hard ML, Wehr AY, Du YC, Weiden PJ, Walling D, von Moltke L. Pharmacokinetic Evaluation of a 1-Day Treatment Initiation Option for Starting Long-Acting Aripiprazole Lauroxil for Schizophrenia. Journal of Clinical Psychopharmacology. 2018;38(5):435-41.

36. Hard ML, Wehr A, von Moltke L, Du Y, Farwick S, Walling DP, et al. Pharmacokinetics and safety of deltoid or gluteal injection of aripiprazole lauroxil NanoCrystalÂ® Dispersion used for initiation of the long-acting antipsychotic aripiprazole lauroxil. Therapeutic advances in psychopharmacology. 2019;9.

37. Mallikaarjun S, Kane JM, Bricmont P, McQuade R, Carson W, Sanchez R, et al. Pharmacokinetics, tolerability and safety of aripiprazole once-monthly in adult schizophrenia: an open-label, parallel-arm, multiple-dose study. Schizophr Res. 2013;150(1):281-8.

38. Potkin SG, Raoufinia A, Mallikaarjun S, Bricmont P, Peters-Strickland T, Kasper W, et al. Safety and tolerability of once monthly aripiprazole treatment initiation in adults with schizophrenia stabilized on selected atypical oral antipsychotics other than aripiprazole. Curr Med Res Opin. 2013;29(10):1241-51.

39. De Filippis S, Cuomo I, Lionetto L, Janiri D, Simmaco M, Caloro M, et al. Intramuscular aripiprazole in the acute management of psychomotor agitation. Pharmacotherapy. 2013;33(6):603‐14.

40. Mauri MC, Reggiori A, Minutillo A, Franco G, Di Pace C, Paletta S, et al. Paliperidone LAI and Aripiprazole LAI Plasma Level Monitoring in the Prophylaxis of Bipolar Disorder Type I with Manic Predominance. Pharmacopsychiatry. 2020;53(05):209-19.

41. Boulton DW, Kollia G, Mallikaarjun S, Komoroski B, Sharma A, Kovalick LJ, et al. Pharmacokinetics and tolerability of intramuscular, oral and intravenous aripiprazole in healthy subjects and in patients with schizophrenia. Clin Pharmacokinet. 2008;47(7):475-85.

42. Mamo D, Graff A, Mizrahi R, Shammi CM, Romeyer F, Kapur S. Differential effects of aripiprazole on D(2), 5-HT(2), and 5-HT(1A) receptor occupancy in patients with schizophrenia: a triple tracer PET study. Am J Psychiatry. 2007;164(9):1411-7.

43. Mizrahi R, Mamo D, Rusjan P, Graff A, Houle S, Kapur S. The relationship between subjective well-being and dopamine D2 receptors in patients treated with a dopamine partial agonist and full antagonist antipsychotics. International Journal of Neuropsychopharmacology. 2009;12(5):715-21.

44. Kim E, Howes OD, Kim BH, Jeong JM, Lee JS, Jang IJ, et al. Predicting brain occupancy from plasma levels using PET: superiority of combining pharmacokinetics with pharmacodynamics while modeling the relationship. J Cereb Blood Flow Metab. 2012;32(4):759-68.

45. Kim E, Howes OD, Turkheimer FE, Kim B-H, Jeong JM, Kim JW, et al. The relationship between antipsychotic D₂ occupancy and change in frontal metabolism and working memory: A dual [¹¹C]raclopride and [¹⁸ F]FDG imaging study with aripiprazole. Psychopharmacology. 2013;227(2):221-9.

46. Yokoi F, Grunder G, Biziere K, Stephane M, Dogan AS, Dannals RF, et al. Dopamine D-2 and D-3 receptor occupancy in normal humans treated with the antipsychotic drug aripiprazole (OPC 14597): A study using positron emission tomography and C-11 raclopride (vol 27, pg 248, 2002). Neuropsychopharmacology. 2002;27(6):1090-.

47. Kegeles LS, Slifstein M, Frankle WG, Xu X, Hackett E, Bae SA, et al. Dose-occupancy study of striatal and extrastriatal dopamine D2 receptors by aripiprazole in schizophrenia with PET and [18F]fallypride. Neuropsychopharmacology. 2008;33(13):3111-25.

48. Ito H, Takano H, Arakawa R, Takahashi H, Kodaka F, Takahata K, et al. Effects of dopamine D2 receptor partial agonist antipsychotic aripiprazole on dopamine synthesis in human brain measured by PET with L-[β-11C]DOPA. PLoS One. 2012;7(9):e46488.

49. Takahata K, Ito H, Takano H, Arakawa R, Fujiwara H, Kimura Y, et al. Striatal and extrastriatal dopamine D₂ receptor occupancy by the partial agonist antipsychotic drug aripiprazole in the human brain: a positron emission tomography study with [¹¹C]raclopride and [¹¹C]FLB457. Psychopharmacology. 2012;222(1):165‐72.

50. Shin S, Kim S, Seo S, Lee JS, Howes OD, Kim E, et al. The relationship between dopamine receptor blockade and cognitive performance in schizophrenia: a [(11)C]-raclopride PET study with aripiprazole. Translational psychiatry. 2018;8(1):87.

51. Kubo M, Koue T, Maune H, Fukuda T, Azuma J. Pharmacokinetics of aripiprazole, a new antipsychotic, following oral dosing in healthy adult Japanese volunteers: influence of CYP2D6 polymorphism. Drug Metab Pharmacokinet. 2007;22(5):358-66.
